# Review of Gastrointestinal Capsule Robots: Materials, Actuation, Diagnostics, and Therapeutics

**DOI:** 10.1002/advs.76800

**Published:** 2026-08-25

**Authors:** Zengsheng Li, Zhongguo Ren, Yiliang Lin, Xianfeng Xia, Tianlong Li, Kai Fung Chan, Philip Wai Yan Chiu, Wei Peng Yong, Mengmeng Sun

**Affiliations:** ^1^ Department of Biomedical Engineering National University of Singapore Singapore Singapore; ^2^ Department of Chemical and Biomolecular Engineering National University of Singapore Singapore Singapore; ^3^ Department of Surgery The Chinese University of Hong Kong Shatin, New Territories Hong Kong SAR China; ^4^ State Key Laboratory of Robotics and System Harbin Institute of Technology Harbin China; ^5^ Suzhou Research Institute of HIT Suzhou China; ^6^ Chow Yuk Ho Technology Centre for Innovative Medicine The Chinese University of Hong Kong Hong Kong SAR China; ^7^ Multi‐Scale Medical Robotics Center Hong Kong Science Park Hong Kong SAR China; ^8^ Li Ka Shing Institute of Health Sciences The Chinese University of Hong Kong Hong Kong SAR China; ^9^ Department of Haematology‐Oncology National University Hospital National University Cancer Institute Singapore Singapore; ^10^ N.1 Institute for Health National University of Singapore Singapore Singapore

**Keywords:** active locomotion, gastrointestinal tract, in situ diagnostic, targeted drug delivery, wireless capsule endoscopy

## Abstract

Gastrointestinal capsule robots are minimally invasive devices for diagnosis and therapy in the digestive tract. This review examines them from a systems engineering perspective, spanning the technological chain from materials and actuation to sensing, treatment, and clinical translation. Structurally, current designs employ rigid polymers and metals, flexible elastomers, and biodegradable or stimuli‐responsive materials to balance mechanical stability, biocompatibility, and environmental adaptability. Locomotion strategies range from passive peristalsis‐driven transit to magnetic, wireless‐powered, multi‐legged, and tethered actuation, paired with magnetic, radiofrequency, vision‐based, MRI‐based, X‐ray‐based, and hybrid localization. Diagnostic capabilities extend from ex situ tissue biopsy and fluid sampling to in situ multimodal sensing and optical imaging for physiological monitoring and biochemical analysis within the gut. Therapeutic developments include targeted drug delivery, microneedle injection, photodynamic therapy, thermal hemostasis, electrical stimulation, metabolic intervention, and mechanical clearance. Despite this progress, challenges persist in actuation capability, energy supply, precise navigation, and system‐level integration. We conclude by highlighting current technological gaps and outlining future directions toward intelligent intraluminal platforms capable of autonomous navigation, multimodal diagnosis, and precision therapy. This work provides a comprehensive reference for advancing the science and application of gastrointestinal capsule robots across multiple fields.

## Graphical abstract

This review examines gastrointestinal capsule robots through a systems engineering lens, covering shell materials, actuation and localization strategies, diagnostic modalities, therapeutic interventions, and commercialization status. By mapping the complete technological chain from fabrication to clinical deployment, this work identifies critical gaps in materials, energy, perception, and system integration that must be addressed to enable autonomous intraluminal diagnosis and treatment.

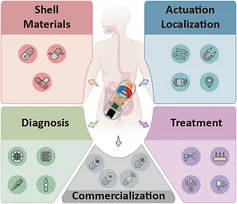

## 1 Introduction

Gastrointestinal (GI) diseases impose a major global health burden [1, 2]. GI malignancies exhibit high incidence and mortality rates globally [3, 4], while non‐neoplastic conditions such as inflammatory bowel disease, GI hemorrhage, and functional gastrointestinal disorders similarly affect a vast patient population [5]. The human digestive tract extends from the esophagus and stomach to the small intestine, colon, and rectum. Each segment has distinct pH conditions, peristaltic patterns, luminal architecture, and mucosal microenvironments [6]. Conventional flexible endoscopy, as one of the most widely employed clinical tools for GI examination and intervention, holds considerable clinical value in performing biopsy, hemostasis, and polypectomy under direct visualization. However, it is still invasive, often requires sedation or anesthesia [7], and depends heavily on operator skill and patient cooperation. In addition, access to deep regions of the GI tract, particularly the small intestine, is still limited [8]. These challenges have driven growing interest in minimally invasive and non‐invasive technologies for GI diagnosis and therapy [9].

**FIGURE 1:**
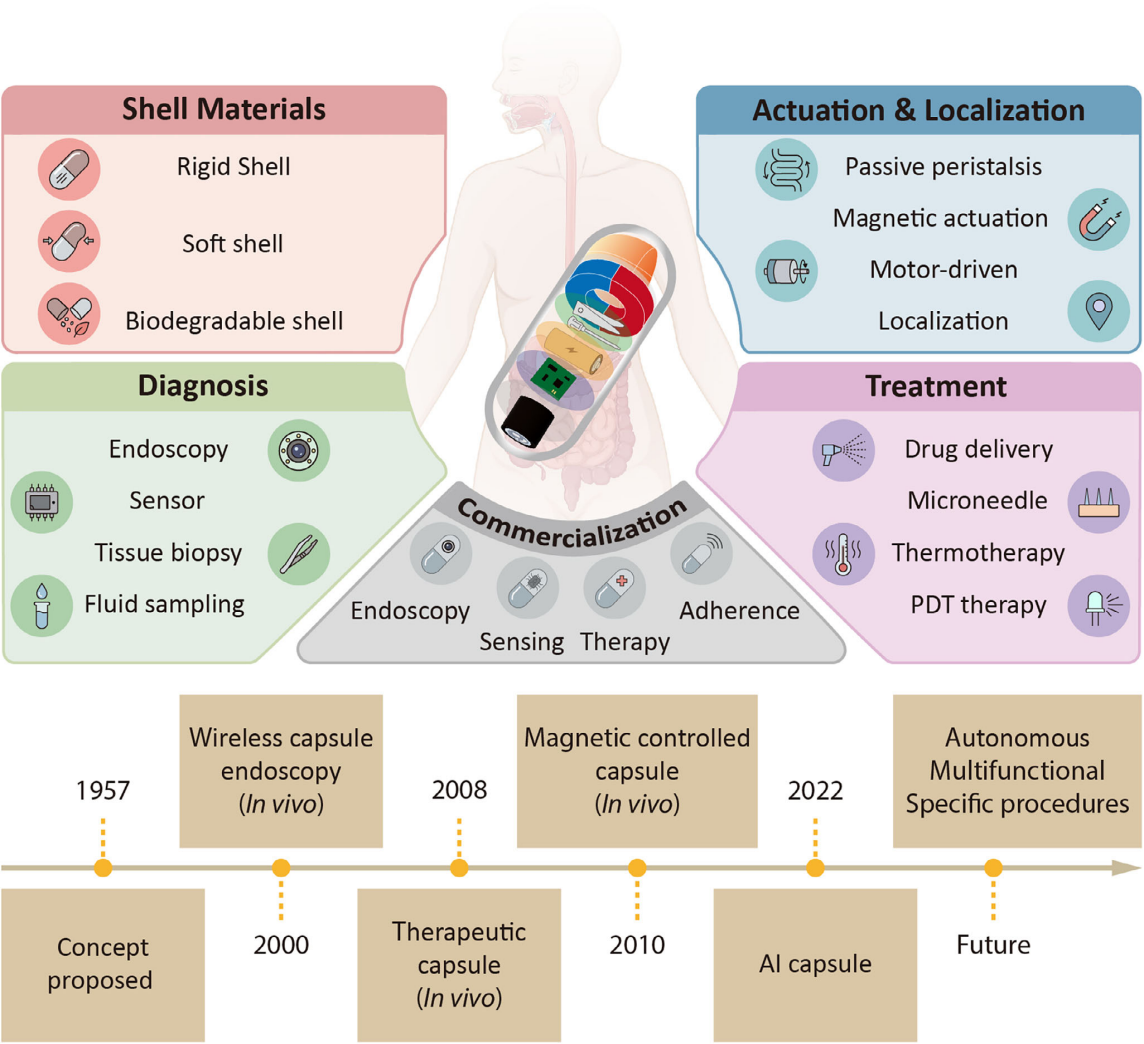
Overview of the research topics and application directions of gastrointestinal capsule robots, including material design, driving and localization, diagnosis, treatment, commercialization, together with a timeline of representative in vivo application milestones. Created by the authors using Adobe Illustrator.

Advances in wireless electronics and device miniaturization have enabled swallowable capsule endoscopes for GI examination [8]. The development of GI capsule robots has spanned nearly seven decades, as summarized in Figure 1. The concept of a swallowable wireless telemetry capsule was proposed in 1957 [10]. The field was transformed in 2000 by the wireless capsule endoscope, which enabled painless imaging of the entire small bowel [8]. Subsequent milestones expanded capsule capabilities from passive imaging toward active intervention and control, including an in vivo therapeutic capsule in 2008 [11], magnetically controlled capsule locomotion in vivo in 2010 [12], and real‐time AI‐assisted lesion detection in vivo in 2022 [13]. Looking ahead, the field is moving toward autonomous, multifunctional capsule robots capable of specific procedures. Early devices were mainly used for luminal visualization, but recent studies have expanded their functions to include environmental sensing, tissue sampling, body fluid analysis, targeted drug delivery, and physical therapy [6, 14]. Their actuation modes have also progressed from passive transport by intestinal peristalsis to active control using magnetic fields, onboard motors, and wireless power [15, 16, 17]. In parallel, capsule designs have moved beyond rigid shells toward flexible and biodegradable stimulus‐responsive materials [18]. Despite this progress, current capsule systems still face major limitations in force output, stable anchoring, real‐time closed‐loop control, and precise tool integration, which restrict their use in surgical‐grade GI interventions [7, 17]. Addressing these limitations will require coordinated advances in materials, actuation, sensing, therapeutic tools, and overall system integration [19]. Several review articles have summarized capsule robots from different perspectives. For example, some focus on actuation and control [15, 16], others on drug delivery system design [20, 21], and others on specific clinical applications [22, 23]. In parallel, recent perspectives on clinical translation have emphasized that moving milli/microrobots into practice requires aligning engineering implementation with unmet clinical needs and value‐centered design [24]. However, a review that takes such an integrated engineering‐implementation perspective on capsule robots is still lacking. In particular, there is a need for a more integrated discussion spanning fabrication materials, actuation and localization, diagnostic and therapeutic functions, commercialization, and future challenges.

This review takes a systems engineering view of capsule robots and examines the field along its main development pipeline. As illustrated in Figure 1, this article is organized around the complete technological chain of capsule robots: Section 2 begins with shell fabrication materials, reviewing the selection, advantages, and limitations of rigid, flexible, and biodegradable materials. Section 3 focuses on driving mechanisms and positioning technologies, encompassing multiple actuation strategies as well as magnetic, radiofrequency, vision‐based, and hybrid localization methods. Section 4 summarizes diagnostic methods and therapeutic strategies, covering ex situ diagnosis, in situ real‐time detection, and multimodal treatment technologies. Section 5 reviews the progress in technology transfer and commercialization. Section 6 analyzes the key challenges and future development directions from the dimensions of materials and structures, energy and actuation, perception and intelligence, and system‐level integration, and highlights the core technological gaps by examining representative pathological scenarios in GI clinical practice that remain beyond the current capabilities of capsule robots. This review aims to provide a practical reference for researchers in the fields of robotics, materials science, and clinical medicine, and to offer a technological perspective for the evolution of capsule robots toward integrated diagnostic‐therapeutic intraluminal intelligent platforms.

## 2 Shell Materials

**FIGURE 2:**
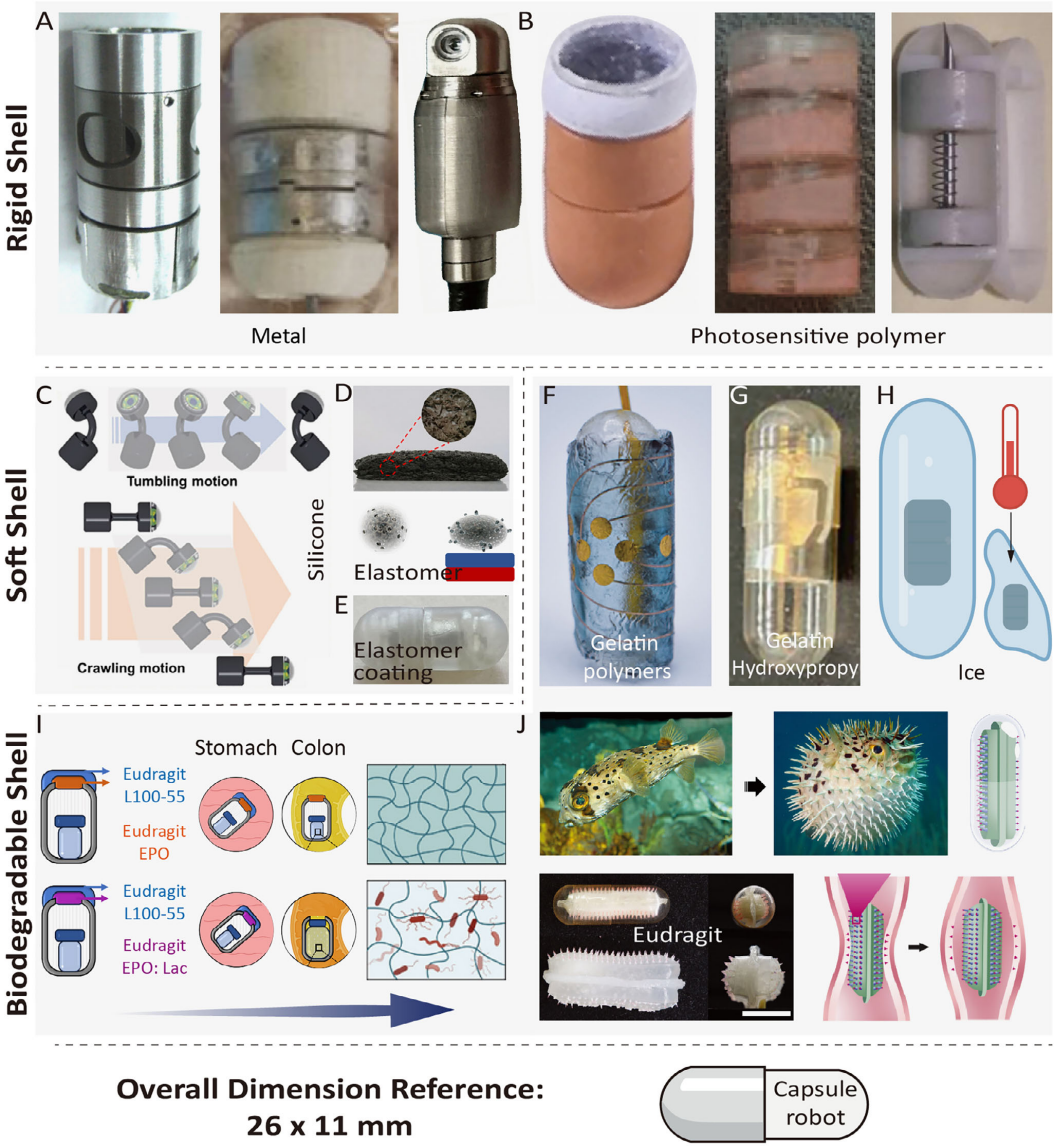
Shell materials and fabrication of gastrointestinal capsule robots, grouped into rigid shells, soft shells, and biodegradable shells. (A) Metallic shells, mainly medical‐grade stainless steel and aluminum alloys, providing high mechanical strength, reliable sealing, and electromagnetic shielding. Reproduced with permission [27, 28, 30]. Copyright 2022, American Association for the Advancement of Science; Copyright 2012, The American Society of Mechanical Engineers; Copyright 2025, Springer Nature. (B) Photosensitive polymer shells based on light‐curable resins, enabling rapid fabrication of complex three‐dimensional structures. Reproduced with permission [32, 33, 34]. Copyright 2025, Oxford University Press; Copyright 2024, MDPI; Copyright 2026, Springer Nature. (C) Silicone soft‐shell capsule embedded with magnetic particles, enabling flexible, compliant deformation. Reproduced with permission [40]. Copyright 2024, Wiley. (D) Hard‐magnetic elastomer foam capsule, in which a porous magnetic elastomer enclosed by an outer elastomer shell deforms under a magnetic field to enable on‐demand drug release. Reproduced with permission [41]. Copyright 2023, American Chemical Society. (E) Elastomer‐coated capsule, in which a soft elastomer layer applied over a rigid shell lowers capsule‐tissue contact pressure during self‐propelled locomotion. Reproduced with permission [44]. Copyright 2023, Springer Nature. (F) Genipin‐cross‐linked gelatin shell, a fully biodegradable material whose mechanical properties and degradation rate are tunable through cross‐linking density. Reproduced with permission [53]. Copyright 2023, Wiley. (G) Gelatin and hydroxypropyl methylcellulose shell for dissolvable encapsulation within the gastrointestinal tract. Reproduced with permission [54]. Copyright 2026, Springer Nature. (H) Ice‐based transient shell that loses structural integrity upon warming to body temperature. Created by the authors using Adobe Illustrator. (I) pH‐responsive Eudragit shells, combining Eudragit L100‐55 with Eudragit EPO or Eudragit EPO:Lac for site‐specific dissolution and triggering between the stomach and the colon. Reproduced with permission [57]. Copyright 2025, Elsevier. (J) Entero‐soluble Eudragit shell that remains intact in gastric acid and dissolves upon reaching the neutral to mildly alkaline environment of the small intestine or colon, enabling site‐specific exposure of internal modules or drug release. Reproduced with permission [58]. Copyright 2024, Springer Nature. The schematic at the bottom indicates the standard capsule robot form factor (26 mm in length and 11 mm in diameter), which serves as a common dimensional reference across most commercial and research prototypes.

The capsule robot shell, which directly interfaces with digestive tract tissue within the standard form factor of approximately 26 mm in length and 11 mm in diameter (Figure 2), serves as the primary physical interface of the system. Beyond encapsulating and protecting the internal microelectronics, sensors, and electromechanical actuation components, the shell also plays a critical role in determining the structural integrity, biocompatibility, tissue–mechanical interaction characteristics, and overall survivability of the system. These requirements arise from the extreme digestive tract environment, including gastric acid corrosion, enzymatic degradation, and mechanical peristaltic compression [6, 25, 26]. Consequently, the selection of shell materials necessitates a systematic balance among functional performance, manufacturing feasibility, and clinical safety. Based on differences in material mechanical properties and functional positioning, existing capsule robot shell solutions can be broadly categorized into three classes: rigid structural materials, flexible and compliant encapsulation materials, and degradable materials (Table 1). Here, the classification between rigid and flexible is not defined by a fixed modulus threshold, but rather by the mechanical relationship between the shell material and the surrounding gastrointestinal tissue. The gastrointestinal wall typically exhibits an elastic modulus on the order of 1 to 10 kPa [18]. Materials whose stiffness exceeds that of the intestinal wall by several orders of magnitude, such as metals and engineering polymers in the GPa range, are classified as rigid, whereas materials whose modulus approaches or overlaps with the tissue range, such as silicone and polyurethane elastomers in the kPa to low MPa range, are classified as flexible [6, 18]. This tissue‐referenced distinction reflects the fundamental design consideration that the mechanical impedance mismatch between the shell and the intestinal wall directly governs contact safety and locomotion compliance.

**TABLE 1:** Representative shell materials for gastrointestinal capsule robots.

Material class	Material category	Representative materials	Principal advantages	Limitations
Rigid	Rigid metals [27, 28, 29, 30]	Medical‐grade stainless steel; aluminum alloy	● High mechanical strength; ● reliable sealing; ● accommodates high‐output‐force actuation	◆ Large mechanical impedance mismatch; ◆ risk of mucosal friction or compression; ◆ fixed dimensions; ◆ non‐degradable with retention risk
	Rigid engineering polymers [31, 32, 33, 34, 35, 36, 37]	Photosensitive resin; polycarbonate; PEEK; epoxy resin	● Rapid light‐curing fabrication of complex structures; ● optical transparency (polycarbonate); ● chemical and acid resistance with biocompatibility (PEEK); ● structural potting and waterproofing (epoxy)	◆ Same impedance mismatch as metals; ◆ fixed external geometry; non‐degradable
Flexible	Flexible silicone elastomers [39, 40, 41, 44]	Silicone elastomer; ultra‐soft silicone; bio‐derived elastomers	● High extensibility and compliance; ● distributes contact stress; ● enables large‐deformation; ● folding; or inflation designs	◆ Low modulus cannot independently sustain high‐load actuation; requires internal support scaffolding; ◆ long‐term chemical stability and anti‐adhesion need validation
	Flexible polyurethane elastomers [42, 43]	Polyurethane elastomer	● Combines mechanical toughness with abrasion resistance; ● suited to repeated deformation and prolonged intestinal‐wall contact	◆ Shares the actuation‐support limitation of flexible materials; ◆ fatigue durability under repeated large deformation needs validation
Degradable	Fully biodegradable materials [53, 54, 55]	Genipin‐cross‐linked gelatin; gelatin; hydroxypropyl methylcellulose; ice (transient)	● Eliminates foreign‐body retention risk; ● no endoscopic retrieval needed; ● degradation rate tunable to module lifespan	◆ Batch‐to‐batch consistency and controllable degradation rate remain bottlenecks; ◆ ice requires demanding fabrication conditions
	Stimuli‐responsive materials [57, 58]	Eudragit enteric polymers (gastro‐soluble and entero‐soluble)	● Site‐specific dissolution exploiting GI pH gradients; ● passive triggering of module exposure or drug release without external control	◆ Partial rather than complete degradation
	Externally triggered degradable materials [59, 60, 61]	Magnetic‐nanoparticle‐doped degradable matrices; electrothermally activated wax and acid‐release layers	● Enable on‐demand shell degradation through external magnetothermal or electrothermal triggering; ● providing active addressability beyond passive pH‐ or enzyme‐driven degradation	◆ Direct use in gastrointestinal capsule shells remains limited; ◆ local thermal safety, degradation controllability, and tissue compatibility require further validation

Rigid materials represent the earliest adopted and currently still predominant category of capsule robot shell designs [7], such as metallic materials and high‐performance engineering polymers. In terms of metallic materials, medical‐grade stainless steel and aluminum alloys, by virtue of their exceptional mechanical strength, reliable sealing performance, and effective electromagnetic shielding capability, are commonly employed in functional capsule prototypes that require the accommodation of high‐output‐force actuation mechanisms, such as motor‐driven rotary cutting modules and spring‐reset puncture devices [27, 28, 29, 30], as shown in Figure 2A. Metallic shells provide a robust structural support framework for complex internal electromechanical systems and are particularly suited to early‐stage prototype validation where high rigidity and dimensional precision are demanded. In terms of engineering polymers, photosensitive resin‐based materials, leveraging light‐curing 3D printing technologies, are capable of rapidly fabricating complex internal channels, miniaturized chambers, and compact assembly structures with high geometric fidelity, as can be seen in Figure 2B, and have become the preferred choice for laboratory‐stage concept verification and design iteration [31, 32, 33, 34]. In addition, engineering polymers including polycarbonate [35], polyetheretherketone (PEEK) [36], and epoxy resin [37] have also been widely adopted across different application scenarios. Polycarbonate offers a favorable combination of optical transparency and impact toughness, making it suitable for imaging‐type capsules requiring optical windows. PEEK, with its outstanding chemical corrosion resistance and biocompatibility, is well suited for prolonged service in corrosive environments such as gastric acid. Epoxy resin is commonly used as a structural adhesive and potting material for internal component fixation and waterproof protection. However, a significant mechanical impedance mismatch exists between rigid shells and the soft intestinal tissue, readily causing mucosal friction injury or localized compression during capsule locomotion. Moreover, the fixed external dimensions are unable to accommodate the dynamically varying luminal geometries across different segments of the digestive tract. Furthermore, these non‐degradable materials must rely on natural excretion or endoscopic retrieval following task completion, posing a potential risk of intracorporeal retention.

To address the mechanical impedance mismatch between rigid shells and the soft intestinal tissue, flexible and compliant encapsulation materials have been progressively introduced into capsule robot shell design [18, 38]. By introducing structural compliance, these materials are capable of effectively distributing contact stresses during capsule‐intestinal wall interaction, substantially reducing mechanical irritation and damage risk to the mucosa. For example, silicone elastomers possess exceptionally high extensibility and compliance, making them particularly suitable for soft‐body capsule shell designs that demand large‐deformation adaptability [39, 40, 41], as illustrated in Figure 2C,D. Polyurethane elastomers combine favorable mechanical toughness with abrasion resistance, making them well suited for applications requiring repeated deformation and prolonged contact with the intestinal wall [42, 43]. As a related strategy, a soft elastomer layer can be coated over a rigid capsule shell to reduce capsule‐tissue contact pressure during locomotion and provide a degree of protection, as illustrated in Figure 2E [44]. In addition, emerging 4D printing materials and advanced fabrication strategies deserve further exploration for capsule robot structures. For example, programmable spatial magnetization stereolithographic printing enables the direct fabrication of thin‐walled soft capsule architectures with spatially encoded magnetization profiles, thereby providing enhanced controllability for magnetic actuation and locomotion [45, 46, 47, 48, 49, 50]. From a functional perspective, flexible materials endow capsule robots with the ability to achieve diameter variation or volumetric expansion, for example through folding or inflation structures that enable safe passage through narrow intestinal segments while deploying in spacious regions such as the gastric cavity to execute diagnostic and therapeutic tasks. Certain designs further adopt a hybrid strategy combining a rigid internal core with a flexible external protective layer, maintaining the structural rigidity of core electromechanical components while utilizing the outer elastomeric layer to achieve compliant interaction with tissue, thereby reconciling functional integration density with tissue compatibility. However, the inherently low mechanical modulus of flexible materials renders them unable to independently sustain high‐load mechanical actuation functions, and the integration of rigid driving components such as motors and gear assemblies typically necessitates additional internal support scaffolding, increasing structural design complexity. Meanwhile, the long‐term chemical stability of elastomeric materials under the acidic and enzymatic conditions of the digestive tract, their fatigue durability following repeated large‐deformation cycles, and the anti‐adhesion capability of flexible surfaces against intestinal mucus and luminal contents all require further validation. Furthermore, although bio‐derived materials possess inherent tissue compatibility advantages, the batch‐to‐batch consistency of their mechanical properties and the precise regulation of controllable degradation rates remain key bottlenecks constraining their engineering‐scale application.

Regardless of whether rigid or flexible composite architectures are employed, non‐degradable shells inevitably face the risks of intracorporeal retention and potential intestinal obstruction following the completion of their intended clinical tasks, a concern that is particularly pronounced in long‐term retentive capsule systems [51, 52]. To fundamentally eliminate the hazards associated with foreign body retention, biodegradable materials have been progressively introduced into capsule robot shell design, emerging as an important technical pathway for achieving a self‐disappearing strategy upon task completion. In terms of fully biodegradable shells, researchers have explored a variety of natural and synthetic degradable materials. Genipin‐cross‐linked gelatin polymers, through the regulation of cross‐linking density, enable precise control over the mechanical properties and degradation parameters of the material within a tunable range, thereby matching the differentiated requirements of various functional modules for structural strength and operational lifespan [53], as shown in Figure 2F. Furthermore, pharmaceutical‐grade materials such as gelatin and hydroxypropyl methylcellulose, which benefit from well‐established industrial processing systems and favorable biosafety records, have been employed to construct shell structures capable of complete dissolution within the digestive tract environment over a preset timeframe [54], as illustrated in Figure 2G. In a more extreme transient material strategy, ice has been explored as a single‐use shell material [55], as can be seen in Figure 2H, leveraging natural melting under body temperature conditions to achieve rapid structural disappearance. Notably, this thermally triggered dissolution mechanism also qualifies ice as a form of stimuli‐responsive material, bridging the boundary between fully biodegradable and environment‐responsive shell strategies. Although this approach still faces significant challenges in terms of fabrication conditions and controllable duration of structural persistence, it provides conceptual inspiration for the zero‐residue robot paradigm. Overall, fully biodegradable materials enable capsule robots to decompose or be absorbed within the body following the completion of diagnostic and therapeutic tasks, thereby eliminating the dependence on endoscopic retrieval or natural excretion. Beyond fully degradable strategies, stimuli‐responsive partially degradable shells constitute another technically important and clinically valuable approach [56]. Such designs do not seek complete structural disappearance, but instead exploit the pH gradient differences across different segments of the digestive tract to achieve selective dissolution of the shell at specific physiological locations, thereby triggering the exposure of internal functional modules or targeted drug release. Represented by the Eudragit family of enteric polymers, two categories of responsive modes can be constructed through different formulations: the gastro‐soluble type dissolves rapidly in the low‐pH strongly acidic gastric environment, while the entero‐soluble type maintains structural integrity in gastric acid and only undergoes dissolution upon reaching the neutral to mildly alkaline environment of the small intestine or colon (Figure 2I,J) [57, 58]. This activation mechanism based on physiological environment self‐triggering provides a passive actuation strategy for capsule robots to execute diagnostic or therapeutic tasks at specific anatomical locations without the need for external control signals. Beyond passive degradation strategies triggered by the gastrointestinal environment, externally controlled degradation may offer capsule robots an additional route toward on‐demand shell disappearance. Magnetic nanoparticles embedded in degradable matrices can generate localized magnetothermal heating under alternating magnetic fields, thereby accelerating degradation of the surrounding matrix [59, 60]. Similarly, electrothermal heating can activate thermally labile materials, as demonstrated by wirelessly powered heaters that melt wax layers and release encapsulated acid to induce rapid degradation of transient electronic components and acid‐sensitive polymer substrates [61]. Although their direct application in gastrointestinal capsule shells remains limited, these magnetic‐ and electrothermal‐triggered mechanisms may provide useful design inspiration for future actively addressable biodegradable shells, provided that local thermal safety is ensured.

From the perspective of future development trends, capsule robot shell design may gradually evolve from a single‐material selection problem toward system‐level co‐optimization of structure and function. The central challenge will likely lie in reconciling competing requirements including mechanical integrity, biocompatibility, environmental responsiveness, and safe excretion within severely constrained device volumes. In this context, materials may no longer function merely as passive structural carriers but instead emerge as active design variables that define functional boundaries and clinical capabilities. Future strategies are expected to increasingly exploit multi‐material hybrid architectures, functionally graded structures, and programmable degradation mechanisms to achieve full‐lifecycle performance. Such approaches could enable coordinated functions including structural support, tissue protection, site‐specific activation, and safe disappearance through the precise spatial integration of rigid, compliant, and degradable components. Ultimately, this deepening coupling between materials engineering and clinical demands may play a critical role in advancing capsule robots from single‐function diagnostic devices toward integrated intraluminal intelligent therapeutic platforms.

## 3 Actuation and Localization

### 3.1 Actuation Mechanism

The driving mechanism constitutes one of the most critical functional modules governing the clinical translation potential of miniature capsule robots, as it fundamentally determines the extent to which these devices can operate within the human body and the breadth of diagnostic and therapeutic procedures they can perform [15, 19]. As the foundational subsystem responsible for enabling autonomous intracorporeal locomotion and functional execution, the driving unit undertakes essential kinematic tasks such as propulsion, steering, and anchoring. It also exerts system‐level influences on the overall energy supply strategy, structural integration complexity, and ultimately the safety profile and biocompatibility of the entire platform. These considerations become particularly acute within the gastrointestinal tract, a highly dynamic physiological environment characterized by complex peristaltic motions, viscous mucus coverage, tortuous and dynamically deforming luminal geometries, and the persistent interference of intraluminal contents [18]. In such a demanding operational milieu, the propulsion efficiency, postural regulation capability, and multi‐degree‐of‐freedom controllability of the driving mechanism collectively dictate whether a capsule robot can transcend the inherent limitations of conventional passive‐transit paradigms to achieve precise navigation, targeted lesion localization, and site‐specific therapeutic intervention. The following subsections review the principal driving strategies developed for capsule robots. Section 3.1.1 addresses tethered actuation schemes, in which a physical cable provides continuous energy supply and signal transmission; Section 3.1.2 then examines untethered configurations, beginning with passive peristalsis‐driven transit and progressing through the major categories of active wireless actuation, including magnetic driving, multi‐legged mechanical propulsion, wireless power‐driven locomotion, electrophysiological coupling, and hybrid mechanisms.

#### 3.1.1 Tethered Capsule Robot

Tethered actuation represents one of the early technical pathways explored in the development of capsule robots. By utilizing a physical cable for energy supply, control signal transmission, and data return, the associated power modules and control units can be partially or entirely externalized, thereby substantially alleviating the integration burden within the capsule interior and enabling more compact form factors with higher power density [62]. However, the introduction of a tether simultaneously imposes constraints on locomotion degrees of freedom and intracorporeal accessibility, while also presenting challenges related to patient comfort and operational complexity [63]. Consequently, tethered driving schemes necessitate a careful balance between enhanced functional capability and clinical feasibility in structural design. Within this framework, a variety of representative tethered capsule robot systems have been developed, employing diverse propulsion mechanisms ranging from simple cable pulling and pneumatic actuation to motor‐driven tracked locomotion and compliant origami‐based structures.

**FIGURE 3:**
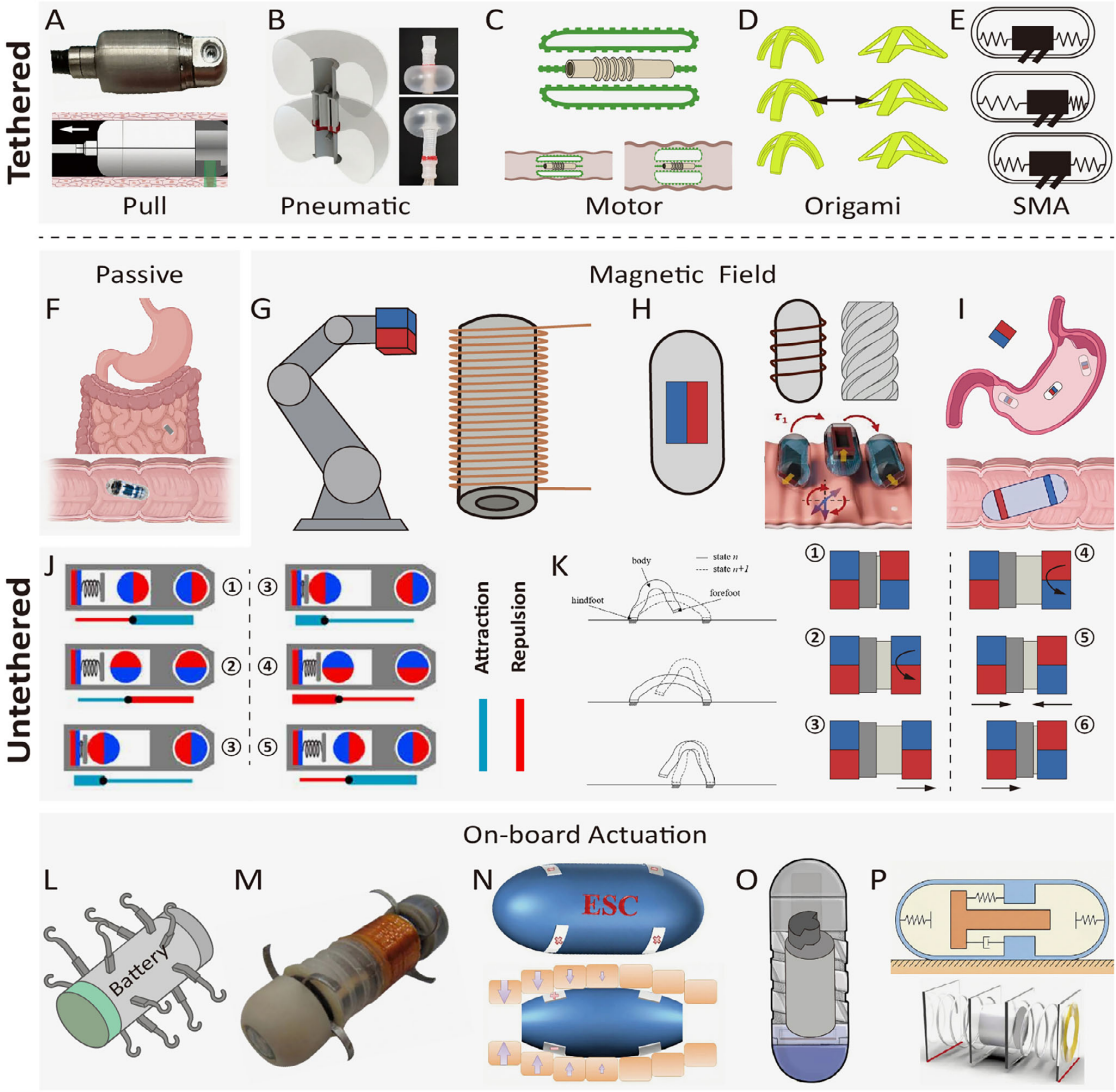
Driving mechanisms of gastrointestinal capsule robots. Current driving strategies can be broadly classified into tethered and untethered modes. (A) Pull‐based locomotion using an external tether. Reproduced with permission [30]. Copyright 2025, Springer Nature. (B) Pneumatic actuation combined with stepwise bioinspired motion. Reproduced with permission [64]. Copyright 2019, Springer Nature. (C) Motor‐driven propulsion using tracked or screw‐like structures, with inflatable adaptation to variable lumen diameters. Created by the authors using Adobe Illustrator, based on the working principle of Ref [65]. (D) Origami‐inspired actuation enabling bidirectional controllable motion through structural deployment and contraction. Created by the authors using Adobe Illustrator, based on the working principle of Ref [66]. (E) SMA‐driven axial locomotion based on thermally induced deformation. Created by the authors using Adobe Illustrator, based on the working principle of Ref [67]. (F) Passive locomotion driven by natural gastrointestinal peristalsis. The upper part was created by the authors using Adobe Illustrator. The lower part was reproduced with permission [14]. Copyright 2024, Elsevier. (G) External magnetic field generation systems based on permanent magnets or electromagnetic coils. Created by the authors using Adobe Illustrator. (H) Rotational magnetic actuation for rolling or helical propulsion. The lower right part was reproduced with permission [73]. Copyright 2024, Springer Nature. The upper right part was created by the authors using Adobe Illustrator, based on the working principle of Ref. [71]. Other part was created by the authors using Adobe Illustrator. (I) Gradient magnetic traction for directional translation. The upper part was created by the authors using Adobe Illustrator. The lower part was reproduced with permission [75]. Copyright 2026, Elsevier. (J) Magneto‐elastic coupling that generates high instantaneous output through cyclic energy storage and rapid release. Reproduced with permission [76]. Copyright 2026, American Association for the Advancement of Science. (K) Inchworm‐like magnetic locomotion based on alternating anchoring and extension. The left part was reproduced with permission [205]. Copyright 2026, OAE. The right part was created by the authors using Adobe Illustrator, based on the working principle of Ref [77]. (L) Onboard multi‐legged actuation using periodic mechanical gait propulsion. Created by the authors using Adobe Illustrator, based on the working principle of Ref [78]. (M) Wireless power‐driven locomotion in which inductive energy transfer powers an internal motor. Reproduced with permission [80]. Copyright 2013, Wiley. (N) Electrophysiological coupling that stimulates intestinal smooth muscle contraction for propulsion. Reproduced with permission [81]. Copyright 2011, Springer Nature. (O) Vibration‐assisted onboard actuation that combines eccentric vibration with other propulsion modes to enhance local disturbance and functional performance. Reproduced with permission [82]. Copyright 2022, American Association for the Advancement of Science. (P) Magnetic–elastic coupling propulsion based on spring energy storage, in which externally supplied magnetic energy is converted into periodic release of elastic potential energy to generate impulsive driving forces. Reproduced with permission [83, 84]. Copyright 2025, Springer Nature; Copyright 2025, American Association for the Advancement of Science.

Li et al. proposed a capsule robot based on a simple pulling mechanism, combined with internal motor rotation to achieve complete luminal imaging [30], as shown in Figure 3A. This approach is relatively straightforward in structure and implementation, yet it is constrained by limited traction range and luminal frictional resistance, making stable clinical application in deep intestinal regions challenging. To achieve more localized motion control, Manfredi et al. adopted a pneumatic actuation approach combined with an inchworm‐like locomotion mechanism to realize stepwise capsule advancement (Figure 3B) [64]. This design is capable of providing relatively controllable axial motion; however, a substantial proportion of the overall system volume is occupied by pneumatic actuators and external tubing components, thereby diminishing the potential advantages of tethered capsules in terms of structural compactness and high‐density functional integration. To accommodate the dynamically varying luminal diameters characteristic of the GI tract, Consumi et al. integrated a caterpillar track structure on the capsule exterior and employed an inflation mechanism to enable dynamic adjustment of the overall outer diameter, thereby adapting motor‐driven propulsion to different intestinal lumen sizes [65], as can be seen in Figure 3C. This system exhibits favorable motion controllability in variable‐diameter environments, yet its dimensional adjustment relies on external manual control and currently lacks real‐time environmental self‐adaptation capability. In the exploration of compliant mechanisms, Ge et al. introduced an origami‐inspired structure onto a conventional passive capsule, achieving bidirectional controllable locomotion through the synergistic interaction between intestinal peristalsis and structural deployment/contraction [66], as illustrated in Figure 3D. This approach demonstrates innovation in directional motion regulation, although its relatively large overall volume limits its applicability in narrow intestinal segments. Additionally, Kim et al. employed shape memory alloy (SMA) as the driving source, combined with an inchworm‐type propulsion structure to achieve axial locomotion [67], as shown in Figure 3E. Despite the relatively simple mechanical configuration of this mechanism, SMA actuation suffers from inherent limitations including slow response speed, high energy consumption, and thermal management difficulties, and consequently related research remains predominantly at an early exploratory stage.

#### 3.1.2 Untethered Capsule Robot

In contrast to tethered configurations, wireless capsule robots represent the predominant architectural paradigm in current capsule endoscopy and robotic capsule research, owing to the elimination of physical tethering that would otherwise constrain natural GI transit and compromise patient comfort [63]. In wireless configurations, all functional subsystems, including imaging, sensing, communication, and in certain designs the driving unit itself, must be accommodated within the highly confined intracapsular volume, imposing stringent constraints on power budget and structural complexity [19]. From a locomotion perspective, wireless capsule robots can be further subdivided according to the source and mode of motive force generation. At one end of the spectrum, passive wireless capsules forgo any active propulsion capability and rely solely on natural peristalsis for transit, prioritizing simplicity and safety at the cost of navigational autonomy. At the other end, active wireless capsules incorporate one or more driving modalities, most commonly external magnetic actuation, to achieve controlled locomotion including propulsion, steering, speed regulation, and regional anchoring. Between these two extremes, a growing number of hybrid schemes have been explored that combine elements of passive transit with selective active intervention, aiming to balance system complexity against clinical functional requirements. The following subsections review these wireless driving strategies, beginning with passive peristalsis‐driven transit and progressing through the major categories of active wireless actuation, including magnetic driving, multi‐legged mechanical propulsion, wireless power‐driven locomotion, electrophysiological coupling, and hybrid mechanisms.

Passive locomotion represents the earliest and most straightforward wireless driving paradigm adopted in capsule robot systems, wherein the capsule relies entirely on the natural peristaltic contractions of the GI tract to complete intraluminal transit [14], as illustrated in Figure 3F. Although this approach inherently precludes active navigation and real‐time postural control, capsules operating in passive mode are generally capable of traversing the entire alimentary canal without sustaining structural damage or inflicting tissue injury, thereby affording a high degree of intrinsic safety. By virtue of this favorable safety profile, the majority of commercially available clinical capsule endoscopes and most function‐specific capsule robots dedicated to sensing tasks continue to employ wireless passive driving as their primary locomotion strategy.

To overcome the fundamental limitations of passive transit, wireless active driving has emerged as a research focus, among which external magnetic field actuation stands out as the most representative and widely adopted technical pathway. This technique exploits the coupling between an externally generated magnetic field and magnetic elements embedded within the capsule body, leveraging magnetic torque and magnetic field gradients to achieve controlled postural deflection and translational propulsion. From a system architecture perspective, external magnetic field generation platforms can be broadly categorized into permanent magnet systems and electromagnetic coil systems [68, 69], as depicted in Figure 3G. Common implementation schemes typically employ multi‐degree‐of‐freedom robotic manipulator arms carrying permanent magnets to enable spatial directional control, or utilize specifically configured electromagnetic coil arrays to synthesize dynamically adjustable complex magnetic fields in real time. In addition, several research groups have pursued hybrid approaches by miniaturizing electromagnetic coils and integrating them at the distal end of a robotic arm, thereby seeking to balance the requirements of a large effective workspace with enhanced local magnetic field control precision [70]. Despite limitations including the substantial physical footprint of external magnetic generation equipment, the nonlinear attenuation of magnetic field strength with increasing distance, and the relatively low spatial resolution, magnetic actuation retains a dominant position in active capsule robot driving research. This is primarily attributed to its fundamentally non‐contact operational principle, the high number of controllable degrees of freedom it affords, and the comparatively mature engineering implementation pathways established through sustained development.

The classical magnetically driven locomotion modes of capsule robots can be broadly classified into rotational driving and gradient traction driving, as schematically illustrated in Figure 3H. In the rotational driving mode, an externally applied rotating magnetic field exerts a magnetic torque on permanent magnets embedded within the capsule, inducing synchronous rotation that translates into rolling or helical propulsion. To address the complex working conditions of narrow and adhesion‐prone intestinal segments, Zhang et al. introduced a helical thread structure on the capsule shell exterior, enabling the device to generate a screw‐like propulsive effect under the influence of a rotating magnetic field [71]. While this design introduces additional geometric complexity to the outer shell surface, it substantially enhances the capsule's ability to traverse constricted intestinal segments by converting rotational motion into directed axial advancement. Building upon this work, Zhou et al. conducted an evaluation of the influence of various helical geometric parameters on propulsion efficiency, thereby establishing both theoretical and experimental foundations for the rational optimization of helical shell architectures [72]. Furthermore, when operating within relatively spacious luminal regions such as the gastric cavity, capsules can exploit rotational motion to achieve wheel‐like wall‐contact rolling locomotion, yielding comparatively high translational velocities and enhanced maneuverability [73]. In contrast, gradient traction driving relies on spatial inhomogeneities in the externally applied magnetic field to exert a direct translational force on the internal magnetic element for directional pulling. However, this modality faces certain safety challenges in the context of clinical translation: the spatial magnetic gradient force attenuates nonlinearly and rapidly with characteristic distance, resulting in severely limited effective driving forces for deep‐tissue applications; moreover, direct magnetic pulling readily risks inducing uncontrolled mucosal tearing or tissue damage. For these reasons, gradient traction is typically confined to liquid‐filled gastric environments, where the surrounding fluid medium serves as a buffering intermediary to enable relatively safe locomotion [74], as shown in Figure 3I. In more constrained environments, such as the intestine, an alternative strategy is to couple magnetic actuation with passive transport. Instead of relying on magnetic forces for continuous propulsion, the device is primarily carried forward by natural intestinal peristalsis, while the magnetic field serves an auxiliary function. In this approach, a weak, nearly uniform magnetic field provides gentle orientation control via magnetic torque, whereas a strong field gradient is applied only locally and transiently to trigger specific functional operations, such as payload release or sample collection, rather than to sustain long‐range pulling [75]. Additionally, the utilization of magnetic levitation to reduce frictional contact between the capsule and the intestinal wall, thereby improving locomotion stability and safety, has also been identified as a promising direction worthy of future exploration.

In recent years, to overcome the limitations of conventional rotational and gradient traction modes in terms of mechanical output and environmental adaptability, researchers have proposed a series of novel magnetically actuated strategies. For instance, Xiang et al. embedded a dual‐permanent‐magnet architecture within the capsule interior, wherein periodic rotation of an external permanent magnet induces the internal magnetic elements to undergo cyclic energy accumulation and instantaneous release, thereby converting continuous external magnetic energy into high‐frequency pulsatile impact forces [76], as can be seen in Figure 3J. Compared with conventional continuous rotational locomotion, this design can generate instantaneous output forces on the order of approximately 3 N, endowing the capsule with the capability to perform high‐payload operations such as tissue puncture, and thus expanding the functional boundaries of magnetically driven capsule robots. Furthermore, to address the slippery and complex physical environment of the intestinal lumen, Yu et al. developed a bioinspired inchworm‐like locomotion structure based on magnetic control principles [77], as illustrated in Figure 3K. This design similarly incorporates two magnets within the capsule body, with their relative rotational positions governed by an external rotating magnetic field. Through the alternating attraction and repulsion between the internal magnets, the capsule emulates a sequential anchoring‐and‐extension mechanism, thereby achieving stable stepwise progression. This biomimetic driving modality demonstrates notable advantages in overcoming luminal slippage and enhancing motion controllability within the GI tract.

Beyond conventional magnetic driving strategies, a number of alternative wireless actuation mechanisms have emerged in recent years, further broadening the locomotion modalities and functional integration capabilities of capsule robots. Valdastri et al. proposed a capsule robot configuration based on multi‐legged gait propulsion, in which an independently battery‐powered capsule module is integrated at the tail end, and stable stepping locomotion is achieved through 12 periodically actuated micro‐legs [78], as shown in Figure 3L. This design enables predictable propulsion through predefined gait patterns and demonstrates a certain degree of gripping and obstacle‐negotiating capability in complex luminal environments. However, the introduction of multi‐degree‐of‐freedom mechanical structures significantly increases system complexity while simultaneously elevating the risks of structural wear and potential tissue damage. Chen et al. integrated a receiving coil within the capsule interior and utilized an external alternating magnetic field to achieve wireless energy transfer, which in turn drives an internal motor to perform inchworm‐like locomotion [79, 80], as illustrated in Figure 3M. This approach combines wireless power delivery with mechanical actuation, thereby alleviating to some extent the constraints imposed by the limited capacity of conventional onboard batteries. Nevertheless, the thermal accumulation effect arising from high‐frequency electromagnetic induction during continuous operation remains a concern that requires further optimization and evaluation. Woo et al. proposed a driving mechanism based on electrophysiological coupling, in which locally applied electrical stimulation induces contraction of intestinal smooth muscle, thereby harnessing the intrinsic peristaltic force of the gut itself to propel the capsule [81], as depicted in Figure 3N. This method reduces the dependence on complex mechanical structures, yet imposes more stringent requirements on the precise regulation of stimulation parameters and long‐term biosafety assessment. Srinivasan et al. employed an eccentric vibration motor as an auxiliary driving source, superimposing centrifugal vibratory output onto an existing magnetically driven helical propulsion architecture [82], as shown in Figure 3O, thereby enhancing localized perturbation capability and providing supplementary mechanical support for functions such as mucosal clearance on tissue surfaces or targeted drug release. In addition, several studies have incorporated spring‐based energy storage structures within the capsule robot body and combined them with embedded magnets to enable external magnetic field energy input, achieving propulsion through the periodic release of elastic potential energy and the conversion of internal collision momentum (Figure 3P) [83, 84]. Such magnetic‐elastic coupling mechanisms offer new perspectives for low‐power, high‐instantaneous‐output driving strategies. If further miniaturization of electromagnetic coils can be realized to enhance energy transfer efficiency and field control precision, these mechanisms hold considerable promise for achieving additional improvements in locomotion stability and energy utilization.

Overall, the driving mechanisms of capsule robots have progressively evolved from early passive transit modes entirely dependent on natural peristalsis toward a diversified technological framework encompassing magnetic actuation, multi‐legged mechanical propulsion, wireless power‐driven locomotion, electrophysiological coupling, pneumatic systems, and various hybrid driving strategies. Different driving paradigms exhibit significant differences in system architecture, energy configuration, and clinical applicability. Tethered configurations, leveraging continuous external power supply and high‐bandwidth signal transmission, are capable of delivering greater actuation force output and more refined real‐time manipulation, though their locomotion degrees of freedom, deep luminal accessibility, and patient compliance are subject to certain limitations. Wireless configurations, by virtue of their superior patient comfort and physically unconstrained intracorporeal accessibility, have become the mainstream direction in current research and application, yet their performance remains fundamentally limited by constrained onboard energy budgets, restricted force output levels, and the engineering challenges of achieving high‐precision multi‐degree‐of‐freedom control within highly compact volumes. Despite substantial progress along both technical pathways, existing driving systems have not yet comprehensively met the demands of complex clinical interventions in terms of output force density, stable navigation precision within tortuous and dynamically deforming luminal environments, tissue‐contact safety margin control, and long‐term operational reliability. Future development trends may increasingly rely on the synergistic integration of high‐power‐density micro‐actuators, intelligent closed‐loop control strategies, and efficient wireless energy transfer or in vivo energy harvesting technologies. Through system‐level optimization across the driving, control, and energy subsystems, it is anticipated that the performance gap between laboratory prototype validation and robust clinical deployment will be progressively narrowed, driving the transformation of capsule robots from diagnosis‐oriented mobile imaging platforms toward intraluminal intelligent operating systems equipped with autonomous navigation and interventional capabilities.

### 3.2 Localization Mechanism

Precise intracorporeal localization constitutes a fundamental prerequisite for capsule robots to achieve targeted diagnostics and therapeutics as well as closed‐loop control. Only when the real‐time position and orientation of the capsule within the digestive tract are accurately determined can precise lesion marking, directional navigation control of the driving system, and spatial positioning of therapeutic operations be realized. However, the digestive tract presents a confined, tortuous, and continuously moving intracorporeal environment. Meanwhile, the complex internal electromagnetic environment, signal attenuation and scattering caused by biological tissue, and the stringent constraints on capsule system volume and power consumption collectively constitute the core challenges in localization system design. Given that the algorithmic principles and system implementations of localization technologies have been elaborated in multiple dedicated review articles [17, 85, 86, 87], this work does not expand upon specific algorithmic details, but instead focuses on providing a comparative overview of existing localization approaches from the perspectives of functional characteristics and clinical applicability, in order to delineate the advantages and technical limitations of different technological pathways. Based on differences in localization signal sources and technical principles, existing capsule robot localization approaches can be broadly categorized into six types: magnetic sensor‐based localization, radiofrequency signal‐based localization, vision‐based localization, magnetic resonance imaging (MRI)‐based localization, X‐ray‐based localization, and multi‐source hybrid localization (Table 2).

**TABLE 2:** Positioning mechanism of gastrointestinal capsule robots.

Localization Category	Advantages	Limitations	Support for Pose Estimation	Reported Localization Accuracy / Resolution
Magnetic sensor‐based [88, 89, 90]	● Unaffected by tissue dielectric properties ● Radiation‐free ● Simultaneous actuation and localization	◆ Susceptible to magnetic interference ◆ Limited effective range ◆ Requires multi‐sensor arrays	Yes (6 DoF, position + orientation).	Tunable 6‐DoF accuracy: 0.04–0.47 mm (position) and 0.15°–2.14° (orientation) [90]
RF‐based [91, 92, 93]	● No additional hardware required ● Leverages existing communication modules ● Low cost	◆ Sensitive to tissue permittivity ◆ Rapid signal attenuation ◆ Severe multipath effects.	Difficult to estimate orientation, requires multi‐antenna arrays for assistance, low accuracy.	Centimeter‐level with conventional RSSI methods, reducible to sub‐millimeter in machine‐learning‐assisted UWB simulations [93]
Vision‐based [94, 95, 96]	● No external equipment required ● Supports concurrent lesion recognition and 3D reconstruction	◆ Demands high illumination and image clarity ◆ Prone to failure in low‐texture environments ◆ Scale drift issues	Yes (camera pose estimation equates to 6 DoF orientation).	0.03 cm displacement error and 0.04 rad error (in roll) [96]
MRI‐based [103]	● Direct anatomical context with high soft‐tissue contrast ● Radiation‐free	◆ Low temporal resolution ◆ Artifacts from magnetic / electronic components ◆ High cost and low accessibility	Limited (position obtainable; orientation constrained by artifacts and temporal resolution).	∼0.46 mm displacement error and 0.48° – 4.40° orientation error
X‐ray‐based [75, 104]	● Clinically mature and widely available ● High temporal resolution ● Large field of view	◆ Ionizing radiation exposure ◆ Only 2D projection (depth/3D orientation hard to resolve) ◆ Low soft‐tissue contrast	Limited (position obtainable; full orientation requires biplane or multi‐view imaging).	Mainly for short‐term image guidance
Hybrid [106, 107, 108, 109]	● Complementary multi‐modal benefits ● Strong anti‐interference capability ● Adaptable to complex environments	◆ High system complexity and power consumption ◆ Requires multi‐source data synchronization and fusion algorithms	Yes (simultaneous estimation of position and orientation achievable via fusion).	5.35 mm position accuracy and 1.46° orientation accuracy (Magnet + IMU) [108] 4.08 mm displacement error and 2.46°orientation error (Magnet + Ultrasound) [109] ……

(Standard deviations are omitted for clarity)

Magnetic sensor‐based localization methods utilize the magnetic field signals generated by permanent magnets embedded within the capsule, measuring the spatial magnetic field distribution through externally arranged magnetic sensor arrays and inverting the three‐dimensional position and orientation of the capsule based on magnetic dipole models, thereby achieving six‐degree‐of‐freedom localization [88, 89, 90]. This approach is unaffected by the dielectric properties of biological tissue and involves no ionizing radiation, offering inherent advantages in terms of safety. For magnetically actuated capsule robots, localization and actuation can share the same magnetic elements, facilitating system simplification and integration. However, magnetic localization is relatively sensitive to external magnetic interference, the effective working range is comparatively limited, and multi‐channel sensor arrays are typically required to ensure localization accuracy, thereby increasing system hardware complexity to a certain extent.

Radiofrequency signal‐based localization methods utilize the wireless signals emitted by the capsule's own communication module, estimating capsule position by measuring parameters such as signal strength or time difference of arrival through external receiving antennas [91, 92, 93]. This approach requires no additional dedicated localization hardware and can directly reuse the capsule's existing data transmission communication link, thereby offering clear advantages in terms of system cost and integration complexity. However, RF signals are significantly affected by variations in dielectric constant when penetrating human tissue, with severe signal attenuation and pronounced multipath effects, resulting in generally low localization accuracy. Furthermore, this method can typically only obtain positional information and has difficulty directly acquiring capsule orientation, with its angular resolution capability remaining relatively limited.

Vision‐based localization methods utilize the onboard camera carried by the capsule, performing feature extraction and matching across consecutive image frames and estimating camera motion trajectories based on visual odometry or simultaneous localization and mapping algorithms, thereby indirectly inferring the position and orientation of the capsule within the digestive tract [94, 95, 96]. This approach requires no additional external equipment support and can share the same image data stream with visual diagnostic functions such as lesion recognition and three‐dimensional reconstruction, offering unique advantages in terms of system functional reuse and data sharing. However, vision‐based localization is highly dependent on illumination conditions and image quality, and is prone to tracking failure in low‐texture regions of the digestive tract or when the lens is obscured by mucus. Concurrently, monocular vision systems also suffer from scale drift issues, making cumulative errors over long‐distance motion difficult to completely avoid.

MR‐based localization methods directly visualize the capsule within its surrounding anatomical context by identifying susceptibility‐induced signal voids, local field distortions, or MRI‐visible contrast markers associated with magnetic components or markers incorporated into the capsule [97, 98, 99]. In contrast to approaches that infer position from signals emitted by or coupled to the capsule itself, MRI can localize the capsule while simultaneously acquiring high‐resolution soft‐tissue images. For specially designed MRI‐compatible magnetic capsule systems, the MRI platform can be used for both imaging/localization and magnetic actuation by exploiting the field gradients generated by the scanner's gradient coils, potentially enabling image‐guided closed‐loop navigation [100, 101, 102, 103, 104]. This capability also makes MRI a useful reference modality for experimental validation of other localization techniques. However, depending on the imaging sequence, the temporal resolution of MRI is generally lower than that of magnetic sensor array or radiofrequency‐based localization, which constrains real‐time tracking of rapidly moving capsules. In addition, magnetic, metallic, or electronic components such as permanent magnets, batteries, antennas, and circuit elements may induce artifacts or other MRI‐related distortions, thereby degrading localization accuracy. The requirement for the capsule's other functional components, such as batteries, antennas, and electronic circuits, to operate reliably within the strong magnetic field and to remain MRI‐conditional further imposes additional constraints on capsule design. Moreover, the high equipment cost, operational complexity, and limited accessibility currently confine this approach mainly to research and validation settings rather than routine clinical capsule localization.

X‐ray‐based localization methods determine the position of the capsule by imaging the radiopaque components or markers it contains, using either single radiographic projections or continuous fluoroscopic imaging. As a clinically mature and widely available modality, radiographic imaging provides a large field of view, while fluoroscopic implementations offer high temporal resolution, allowing the capsule to be tracked in real time against recognizable skeletal and gross anatomical landmarks. Single abdominal radiographs combined with radiopaque markers have long been used in gastrointestinal transit studies [105], whereas fluoroscopy has been used for the real‐time guidance of magnetically actuated capsules during navigation and intervention, particularly in experimental or preclinical settings [75, 104]. Compared with magnetic sensor array or radiofrequency‐based approaches, this modality provides direct projection‐based anatomical context and does not require active signal emission from the capsule or electromagnetic coupling‐based signal interpretation. However, X‐ray imaging exposes the patient, and during fluoroscopic procedures potentially also the operator, to ionizing radiation, which limits the permissible imaging duration and frequency and restricts its suitability for prolonged or repeated tracking. In addition, conventional planar fluoroscopy provides only two‐dimensional projection information, making it difficult to resolve the depth position and full three‐dimensional orientation of the capsule without biplane systems, multi‐view imaging, additional sensors, or reconstruction algorithms. The relatively low soft‐tissue contrast of X‐ray imaging also constrains its ability to localize the capsule relative to specific mucosal lesions, so it is often combined with other localization or imaging techniques in practice.

To overcome the limitations of individual localization modalities, hybrid localization approaches achieve complementary enhancement and robust positioning through the fusion of multi‐source information from magnetic fields, radiofrequency signals, and vision [106, 107, 108, 109]. Multi‐source fusion methods are capable of exploiting complementary information across different localization modalities, offering clear advantages in anti‐interference capability and environmental adaptability, and are particularly suited to actively driven capsule robots requiring high‐precision closed‐loop control. However, multimodal fusion correspondingly increases system complexity and power consumption demands, while also requiring solutions for temporal synchronization of multi‐source data and efficient fusion algorithms, imposing higher demands on the limited computational resources available within the capsule.

## 4 Diagnosis and Treatment

### 4.1 Diagnostic Method

Disease diagnosis constitutes one of the core application‐driven forces behind the technological development of capsule robots. Compared with conventional endoscopy, which is highly dependent on operator expertise and frequently accompanied by instrument‐induced friction and traction discomfort, capsule robots offer distinct advantages including non‐invasiveness, elimination of anesthesia requirements, and superior patient comfort, enabling functional assessment of the GI tract while substantially reducing the physiological burden on patients [7, 19]. From the perspective of diagnostic pathways, capsule robot‐based disease detection can be categorized into two principal modalities. The first is ex situ diagnosis, wherein onboard miniaturized biopsy or fluid collection mechanisms are employed to actively acquire tissue or biochemical specimens at targeted lesion sites and safely transport them out of the body for subsequent high‐precision histopathological and laboratory examination outside the GI tract. The second is in situ diagnosis, which relies on densely integrated optical imaging modules, chemical sensors, or biosensors within the capsule to directly perform physiological parameter measurement, real‐time extraction of pathological features, and wireless transmission of diagnostic data at the site of interest within the living GI environment, without requiring physical retrieval of specimens.

#### 4.1.1 Ex Situ Diagnosis

**FIGURE 4:**
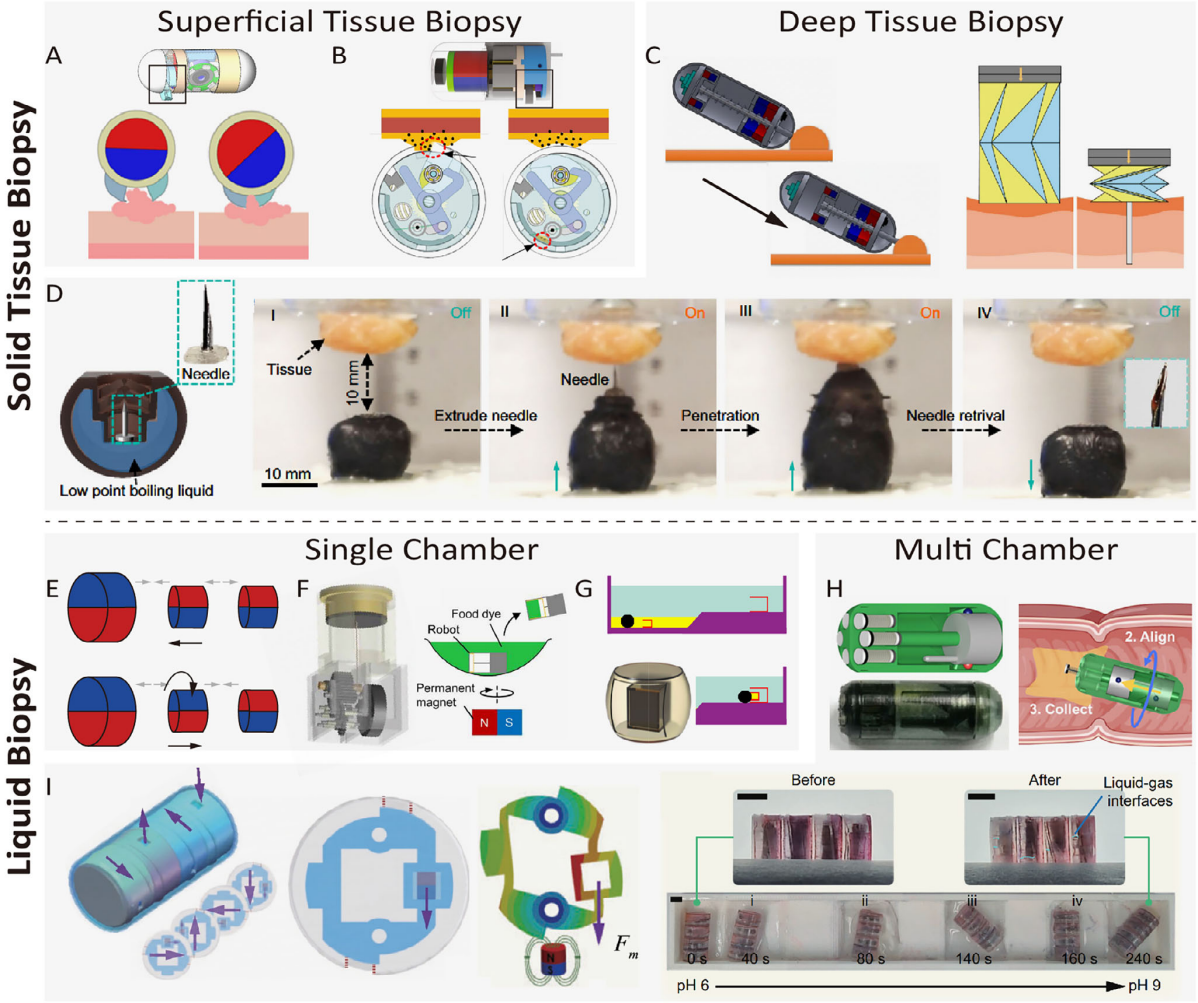
Tissue biopsy and liquid sampling by gastrointestinal capsule robots. (A) Magnetically triggered forceps‐based tissue biopsy, in which an external magnetic field controls forceps deployment and retraction. Reproduced with permission [110]. Copyright 2017, MDPI. (B) Rotary cutting or scraping biopsy driven by a spiral spring energy‐storage mechanism for superficial tissue sampling. Reproduced with permission [27]. Copyright 2022, American Association for the Advancement of Science. (C) Needle‐based deep tissue biopsy designs, including spring‐reset, flexure‐hinge, and kirigami bistable configurations. Reproduced with permission [33, 113]. Copyright 2024, MDPI; Copyright 2025, Elsevier. (D) Thermally driven needle biopsy based on phase transition of a low‐boiling‐point liquid, enabling needle extrusion, tissue penetration, and retrieval. Reproduced with permission [114]. Copyright 2024, Springer Nature. (E) Single‐chamber intestinal fluid sampling based on a magnetic spring mechanism. Created by the authors using Adobe Illustrator, based on the working principle of Ref [116]. (F) Liquid aspiration driven by a micro‐gearbox. Reproduced with permission [117]. Copyright 2022, American Association for the Advancement of Science. (G) A dual‐layer liquid‐sampling capsule in which magnetically controlled inner‐shell motion enables sampling. Reproduced with permission [118]. Copyright 2024, National Academy of Sciences. (H) A multi‐chamber capsule for region‐specific collection and isolated storage of luminal fluid or microbiome samples. Reproduced with permission [119]. Copyright 2025, Wiley. (I) A highly integrated multi‐chamber array capsule with controllable valve structures for independent multi‐site sampling and sample isolation. Reproduced with permission [32]. Copyright 2025, Oxford University Press.

Tissue biopsy remains the most direct and reliable approach for examining GI tract pathologies, making the integration of biopsy functionality into capsule robots an important research direction. Among the various biopsy mechanisms explored for capsule robot platforms, forceps‐based tissue acquisition represents the most commonly adopted scheme. Le et al. developed a miniaturized gripper‐based biopsy module for the capsule endoscope, in which an internal permanent magnet, driven by an external rotational magnetic field, rotates to actuate the cutting blades through a gear train [110], as shown in Figure 4A. Although this design successfully replicates the grasping and sampling function in a structurally compact form, the miniaturized forceps frequently encounter a gripping force bottleneck imposed by the stringent spatial constraints and limited actuation output inherent to the capsule platform; this not only restricts sampling depth to the superficial mucosal layer in most cases, but also readily leads to sampling failure due to inadequate grip retention. To circumvent the issue of insufficient output force leading to sampling failure, rotary cutting‐based sampling mechanisms have been proposed [27], as illustrated in Figure 4B. Song et al. integrated a clockwork‐type elastic energy storage device within the capsule, releasing stored potential energy to drive high‐speed rotational blade cutting, thereby achieving substantially enhanced instantaneous output forces. Zhao et al. introduced a kirigami configuration, combining controllable deployable spikes with a rotary scraping mechanism to accomplish tissue detachment [111]. These cutting and scraping mechanisms offer advantages in acquiring tissue specimens with larger surface areas, yet their effective range remains confined to the tissue surface, rendering them unable to access and retrieve deep‐seated pathological tissue beneath the submucosal layer. To overcome the physical limitations of deep‐layer sampling, needle puncture structures have been further incorporated into capsule systems, as can be seen in Figure 4C. Rashid designed a spring‐reset needle biopsy capsule that relies on an external magnetic field to drive linear translation of an internal magnet for deep tissue puncture, followed by automatic retraction via the elastic restoring force of the spring upon removal of the magnetic field [33]. To enhance the guidance precision of miniaturized puncture mechanisms, Son et al. proposed a biopsy structure based on flexure hinges and employed a Sarrus linkage mechanism to assist motion reset, thereby improving the axial stability of both the puncture and retrieval processes and mitigating issues such as friction and jamming to a certain extent [112]. Huang et al. further designed the outer shell as a kirigami bistable structure, utilizing structural state transitions to facilitate puncture actuation and reset assistance [113]. However, such bistable designs typically correspond to a fixed mechanical stroke, rendering the puncture depth non‐adjustable and potentially increasing the risk of unintended tissue injury. Furthermore, in the domain of non‐conventional actuation, Hao et al. proposed a novel thermally driven biopsy mechanism [114], as depicted in Figure 4D. This system injects a low‐boiling‐point liquid into a sealed capsule cavity and employs externally applied high‐intensity focused ultrasound to induce heating, vaporization, and volumetric expansion of the liquid, utilizing the resulting gas pressure to drive the puncture needle into the tissue, followed by passive needle retrieval through natural cooling and liquid contraction. This magnet‐free actuation approach confers MRI compatibility upon the capsule robot; however, the external ultrasound energy transmission system is structurally complex, and significant challenges remain regarding thermal control precision during the phase‐transition process as well as thermal safety for the surrounding intestinal tissue, with the current state of development remaining at the proof‐of‐concept stage.

Beyond tissue biopsy, body fluid analysis also holds significant value in GI disease diagnosis. Digestive tract fluids contain abundant metabolic products, inflammatory mediators, and microbial information, making the development of capsule robots capable of actively aspirating intraluminal fluid samples and safely transporting them out of the body for subsequent analysis an important research direction in ex situ diagnostics [115]. Zhang et al. arranged multiple small permanent magnets within the capsule interior to construct a magnetic spring mechanism, which triggers spring release by altering the strength and direction of the external magnetic field, thereby generating negative pressure within the internal chamber to achieve intestinal fluid aspiration [116], as shown in Figure 4E. This structural design is compact, yet constrained by the limited output force of the magnetic spring, the system frequently struggles to guarantee consistent aspiration volume and sampling reliability when encountering high‐viscosity or low‐fluidity intestinal fluids. To enhance output capability, Hong et al. employed a micro gearbox as the driving unit, amplifying output torque through a multi‐stage gear reduction mechanism to strengthen the system's aspiration and pumping capacity for high‐viscosity liquids [110, 117], as depicted in Figure 4F. Although this approach effectively improves output performance, the introduction of a multi‐stage transmission chain also increases manufacturing complexity. In terms of system architecture innovation, Sun et al. further proposed a dual‐layer inner‐outer shell micro liquid sampling capsule [118], as can be seen in Figure 4G. This design utilizes a magnetic fluid robot to actuate the inner shell, accomplishing both locomotion and sampling operations, and demonstrates notable advantages in structural modularity and functional flexibility. It is worth noting that the aforementioned approaches are largely limited to single‐chamber sampling configurations, which cannot fulfill the clinical requirement for multi‐point, multi‐region independent sampling across different physiological segments of the digestive tract. To address this limitation, Park et al. developed a capsule robot integrating multiple independent fluid storage chambers [119], as illustrated in Figure 4H, enabling regionalized fluid collection and isolated storage by sequentially opening corresponding chambers at different spatial positions. To further simplify the structure and control methodology of multi‐chamber designs, Wu et al. proposed a highly integrated multi‐chamber array architecture, in which multiple physically isolated fluid storage units are bonded together to form a complete capsule robot, with flexible hinges and small magnets employed to construct directionally selective magnetic valve structures [32]. Only when an oscillating magnetic field of a specific orientation is externally applied does the corresponding chamber respond precisely and open independently, as shown in Figure 4I, thereby enabling controllable multi‐region fluid sampling. In parallel, Dong et al. developed millimeter‐scale soft capsules integrating flexible magnetic valves and a superabsorbent polymer for wireless, on‐demand liquid sampling and sealing in confined fluidic environments, demonstrating image‐guided magnetic navigation and sampling in phantoms and ex vivo animal organs and thereby extending active fluid sampling toward narrow digestive‐tract‐associated lumens such as the bile and pancreatic ducts [120].

Overall, capsule robots for ex situ diagnosis have progressively established a relatively complete technological framework along both the tissue biopsy and body fluid collection directions. In the domain of tissue biopsy, from forceps‐based sampling and rotary cutting structures to needle puncture mechanisms, the various approaches demonstrate a technological evolution trajectory of increasing depth in terms of structural complexity, force output capability, and achievable sampling depth. Meanwhile, beyond conventional motor or magnetic actuation, the introduction of thermal actuation and other novel micro‐actuation principles has further enriched the repertoire of driving modalities and enhanced the flexibility and compatibility of system integration. In the domain of body fluid collection, system design has progressed from early single‐chamber aspiration structures toward multi‐chamber independent sampling arrays with isolated storage architectures, with the research focus expanding from single‐point fluid acquisition to regionalized sampling and independent sample preservation across different segments of the entire digestive tract. However, existing ex situ diagnostic approaches still face several common challenges. First, how to achieve stable, controllable, and safe force output while ensuring sufficient sampling depth remains a core difficulty for biopsy mechanisms, with particularly notable deficiencies persisting in the reliable acquisition of submucosal and deeper pathological tissues. Second, the aspiration efficiency, consistency, and anti‐cross‐contamination capability of fluid sampling systems in high‐viscosity intestinal fluid environments still require further optimization. Future breakthroughs are likely to concentrate on two fronts: first, combining high‐force‐output micro‐actuation mechanisms with real‐time force feedback to realize intelligent biopsy systems with adjustable sampling depth and controllable tissue damage; second, constructing environmentally self‐adaptive multi‐channel sampling architectures capable of dynamically adjusting sampling strategies according to the physiological characteristics of different digestive tract segments, thereby better serving the demands of precision medicine for regionalized and biologically differentiated information acquisition.

#### 4.1.2 In Situ Diagnosis

In contrast to ex situ diagnostic approaches that require physical extraction of tissue or fluid specimens, in situ diagnosis relies on densely integrated sensors and imaging modules within the capsule to directly perform physiological parameter measurement, pathological feature extraction, and wireless data transmission at the site of interest within the living GI environment [14, 25]. This diagnostic paradigm eliminates the need for sample retrieval and subsequent laboratory analysis, enabling immediate acquisition of diagnostic information during capsule transit. Based on the nature of the acquired signals, in situ diagnostic capabilities of capsule robots can be broadly divided into two categories: sensor‐based physiological and biochemical detection, and optical imaging‐based visual inspection. The former encompasses a range of sensing modalities targeting physical, chemical, and biological parameters of the GI microenvironment, while the latter leverages advanced optical technologies to achieve enhanced tissue visualization, spectral contrast improvement, and three‐dimensional structural reconstruction.

**FIGURE 5:**
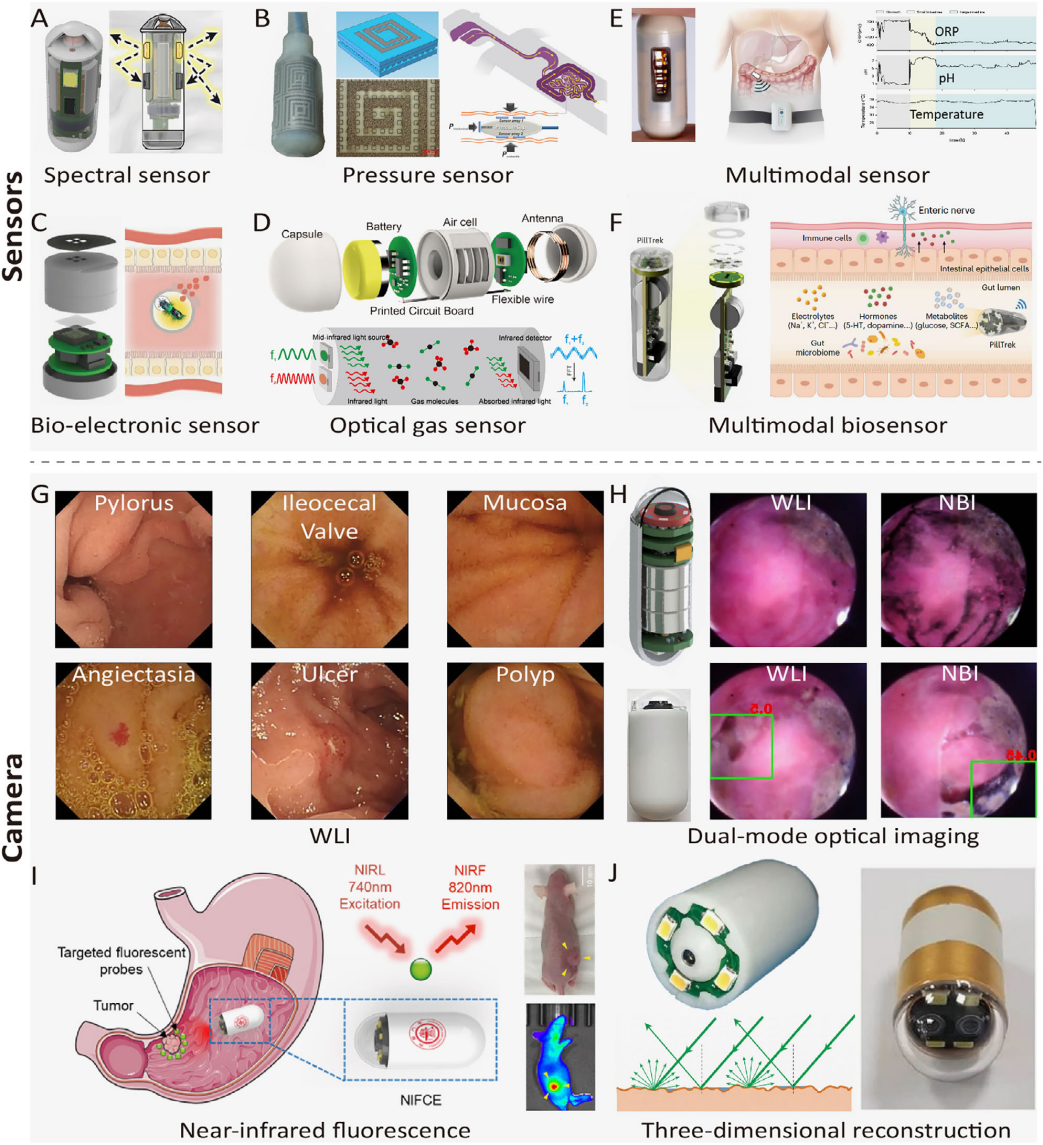
In situ diagnosis of gastrointestinal capsule robots: sensing and vision. (A) A capsule robot integrating white‐light LEDs and multichannel spectral sensors for tissue‐state assessment and ischemia diagnosis by analyzing reflected light intensity across different wavelength bands. Reproduced with permission [121]. Copyright 2025, American Association for the Advancement of Science. (B) A capsule robot with a flexible pressure sensor array for monitoring gastrointestinal pressure distribution through capacitance‐ and resonance‐frequency changes induced by peristaltic compression. Reproduced with permission [122]. Copyright 2024, Elsevier. (C) A bioelectronic hybrid sensing capsule that uses engineered bacterial strains to generate bioluminescent responses for in situ detection of chemical molecules such as NO and H_2_O_2_. Reproduced with permission [123]. Copyright 2023, Springer Nature. (D) A capsule robot with a miniaturized optical gas‐sensing chamber for quantitative detection of characteristic gastrointestinal gases based on infrared absorption. Reproduced with permission [124]. Copyright 2026, Elsevier. (E) A capsule robot integrating oxidation‐reduction potential, pH, and temperature sensors for continuous monitoring of gastrointestinal microenvironmental changes. Reproduced with permission [36]. Copyright 2025, Springer Nature. (F) A capsule robot incorporating a miniaturized wireless electrochemical workstation and multiple recognition elements for simultaneous monitoring of metabolites, hormones, ions, and pH. Reproduced with permission [125]. Copyright 2025, Springer Nature. (G) A capsule endoscope employing white‐light imaging (WLI) for faithful reproduction of natural tissue color and visualization of mucosal surface morphology, serving as the fundamental imaging modality for preliminary gastrointestinal screening. Reproduced with permission [127]. Copyright 2025, Springer Nature. (H) A dual‐mode optical imaging capsule integrating both white‐light imaging and narrow‐band imaging (NBI) within a single system, utilizing narrow‐band light at specific wavelengths to enhance microvascular contrast and improve visualization of early‐stage lesions. Reproduced with permission [13]. Copyright 2025, Springer Nature. (I) A near‐infrared fluorescence capsule endoscope incorporating a white‐light source, a near‐infrared light source, an image sensor, and a band‐rejection filter, enabling simultaneous acquisition of white‐light and near‐infrared fluorescence images for targeted detection of deep or occult micro‐lesions. Reproduced with permission [128]. Copyright 2024, Elsevier. (J) Three‐dimensional depth reconstruction approaches for capsule endoscopy, including photometric stereo methods that analyze reflectance characteristics under multiple illumination directions to compute surface normals, and binocular stereo vision systems that integrate dual miniaturized cameras for disparity‐based three‐dimensional reconstruction and direct measurement of lesion dimensions. Reproduced with permission [129, 130]. Copyright 2020, MDPI;Copyright 2020, Springer Nature.

Sensor‐based physiological and biochemical detection constitutes the first major category of in situ diagnostic capabilities, aiming to quantitatively characterize the physical, chemical, and biological state of the GI microenvironment through integrated onboard sensing elements. Chen et al. integrated multiple sets of white‐light light‐emitting diodes and multi‐channel spectral sensors onto a capsule robot [121], as shown in Figure 5A. This system emits white light toward the intestinal wall and collects the reflected optical signals, subsequently measuring light intensity across 10 distinct wavelength bands. Combined with a brightness‐index‐based evaluation algorithm capable of reflecting tissue status, real‐time diagnosis of acute mesenteric ischemia was achieved. In the domain of GI motility monitoring, Mitrakos et al. integrated a flexible pressure sensor array onto the capsule robot surface [122], as illustrated in Figure 5B. When the capsule is mechanically compressed by GI peristaltic contractions, deformation of the internal microstructures within the sensors induces capacitance changes, which in turn cause a measurable shift in the resonant frequency wirelessly read out by an external antenna. This wireless mechanical readout mechanism enables real‐time monitoring of multi‐point pressure distribution along the GI tract. However, the aforementioned systems are primarily focused on the detection of a single physiological or physical parameter. To achieve in situ sensing of the chemical microenvironment, Inda‐Webb et al. proposed a bio‐electronic hybrid sensing capsule that incorporates multiple groups of engineered bacterial strains, each specifically responsive to different chemical molecules, within the capsule robot [123], as depicted in Figure 5C. Upon activation, these strains generate bioluminescent signals of varying intensity depending on the concentration of target molecules and exposure duration, which are subsequently detected by a photoelectric conversion circuit for quantitative analysis of target biomarker concentrations. This system is capable of continuously monitoring readily degradable molecules such as NO and H_2_O_2_ at relatively low power consumption. For metabolic monitoring of signature gases within the digestive tract, Wu developed a highly integrated miniaturized optical gas sensing chamber [124], as can be seen in Figure 5D. GI gases enter the sensing chamber through a PDMS membrane, whereupon frequency‐modulated infrared probe light at a specific wavelength undergoes intensity attenuation through absorption by target gas molecules as it traverses the gas sample. The signal is received and demodulated by a photodetector, thereby enabling quantitative detection of multiple gas concentrations. This system can monitor gas concentration variations in situ and capture dynamic metabolic transformations in real time. Furthermore, Even et al. integrated an oxidation‐reduction potential sensor, a pH sensor, and a temperature sensor within a capsule robot [36], as shown in Figure 5E. By measuring the voltage difference between a working electrode and a reference electrode exposed to digestive fluids, this capsule robot enables direct measurement of the redox state of GI contents. The device can continuously monitor redox dynamic changes throughout the entire digestive tract transit, thereby providing a more comprehensive characterization of the GI microenvironment evolution. Building further upon this concept, Min et al. integrated a miniaturized wireless electrochemical workstation within a capsule robot and constructed a sensor array incorporating multiple specific recognition elements including selective membranes, aptamers, and enzymes (Figure 5F) [125]. Upon binding of target biochemical molecules with their corresponding recognition elements, the resulting signals are transduced into readable electrical outputs through potentiometric, amperometric, or voltammetric detection techniques, enabling simultaneous measurement of multiple biomarker concentrations. This system is capable of continuous, real‐time monitoring of dynamic changes in metabolites and hormones within the GI tract, including serotonin, glucose, pH, and ionic strength, demonstrating the potential of multimodal electrochemical biosensing for in situ detection.

While the sensor‐based approaches described above enable quantitative measurement of specific physiological and biochemical parameters, they inherently lack the ability to provide spatially resolved visual information regarding tissue morphology and surface architecture. For comprehensive in situ diagnosis, direct optical visualization of the mucosal surface remains indispensable, as it allows clinicians to identify structural abnormalities, vascular pattern alterations, and subtle color changes that are indicative of early‐stage pathologies. Accordingly, substantial research efforts have been devoted to advancing the optical imaging capabilities of capsule robots, progressing from conventional white‐light imaging toward spectrally enhanced, molecularly targeted, and three‐dimensionally resolved imaging modalities.

White‐light imaging (WLI) is capable of faithfully reproducing the natural color of tissue and clearly presenting morphological changes on the mucosal surface, and therefore remains the fundamental imaging modality for preliminary GI screening and clinical diagnosis in current capsule endoscopy [126, 127], as shown in Figure 5G. However, conventional WLI still exhibits certain limitations in terms of tissue contrast and structural resolution capability: its contrast is relatively limited, making it difficult to highlight subtle structural differences; the optical penetration depth is shallow, rendering subsurface mucosal lesions difficult to observe; and the absence of depth information constrains the quantitative assessment of lesion size and morphology. To overcome the limitations of tissue contrast, researchers have progressively introduced spectrally enhanced imaging techniques. Sahafi et al. proposed a dual‐mode optical imaging capsule that simultaneously integrates WLI and narrow‐band imaging (NBI) within a single system [13], as illustrated in Figure 5H. Compared with WLI alone, NBI utilizes narrow‐band light at specific wavelengths to enhance hemoglobin absorption characteristics, thereby improving the contrast between microvascular structures and surrounding tissue and enabling clearer visualization of early‐stage lesion areas. Building upon this foundation, molecular‐targeted fluorescence imaging has further extended the diagnostic capabilities of capsule robots. Zhou et al. developed a near‐infrared fluorescence capsule endoscope by integrating a white‐light source, a near‐infrared light source, an image sensor, and a band‐rejection filter within a single capsule, enabling simultaneous acquisition of WLI and near‐infrared fluorescence images [128], as depicted in Figure 5I. This system can be used in conjunction with exogenous targeted fluorescent probes to perform specific fluorescent labeling and imaging of deep or occult micro‐lesions, thereby significantly enhancing the detection sensitivity for pathologies that are difficult to identify under conventional WLI. Beyond two‐dimensional spectral information, the three‐dimensional spatial structure of digestive tract tissue surfaces also holds significant importance for lesion identification and quantitative assessment, as can be seen in Figure 5J. Hao et al. proposed a depth reconstruction method based on photometric stereo, which illuminates the target surface from multiple lighting directions and analyzes the reflectance characteristics under different illumination conditions using specular and diffuse reflection models, thereby computing surface normals and reconstructing depth information [129]. An alternative approach is based on binocular vision principles, wherein Nam et al. integrated two miniaturized cameras within a capsule robot and employed stereo matching algorithms to calculate disparity, enabling three‐dimensional reconstruction of digestive tract structures and direct measurement of lesion dimensions [130].

Overall, in situ real‐time diagnostic capabilities of capsule robots have advanced substantially across both sensor‐based detection and optical imaging domains. In the sensing dimension, the field has progressed from single‐parameter physical measurements toward multimodal platforms capable of simultaneously monitoring mechanical, chemical, gaseous, electrochemical, and biological indicators, providing increasingly comprehensive characterization of the GI microenvironment. In the imaging dimension, the evolution from conventional WLI toward NBI, near‐infrared fluorescence imaging, and three‐dimensional depth reconstruction has progressively enhanced tissue contrast, detection sensitivity for occult lesions, and spatial quantification capability. However, several challenges remain. Most sensor systems still operate along a single sensing modality, and the integration of heterogeneous sensor arrays within the severely constrained capsule volume continues to pose significant engineering difficulties. In terms of imaging, the trade‐off between image resolution, field of view, and power consumption has not been fully resolved, and real‐time onboard image processing capability remains limited by computational and energy constraints. Future development is expected to converge toward the deep integration of multimodal sensing and advanced imaging within a unified capsule platform, coupled with onboard artificial intelligence algorithms for real‐time lesion detection and classification, ultimately enabling capsule robots to serve as autonomous, comprehensive diagnostic instruments capable of replacing or complementing conventional endoscopic examination.

### 4.2 Treatment Method

Building upon the foundation of disease diagnosis and lesion localization, achieving active therapy and precision drug delivery represents a critical direction in the evolution of capsule robots toward integrated diagnostic‐therapeutic platforms. Compared with conventional oral drug administration that relies on passive diffusion or systemic absorption, capsule robots are capable of accomplishing targeted, quantitative, and on‐demand release within the GI lumen, thereby enhancing local drug concentrations, reducing systemic side effects, and expanding the application scope of biologics and other pharmaceuticals that are difficult to absorb through conventional oral routes [131]. Guided by the objectives of precision delivery, controllable triggering, and sustained release, researchers have proposed a variety of innovative structural and actuation mechanisms. Beyond drug delivery itself, the therapeutic repertoire of capsule robots has further expanded toward multimodal physical interventions, including electrophysiological modulation, photodynamic therapy, and mechanical clearance, progressively establishing a diversified intraluminal treatment framework.

#### 4.2.1 Targeted Intraluminal Drug and Biologic Delivery

**FIGURE 6:**
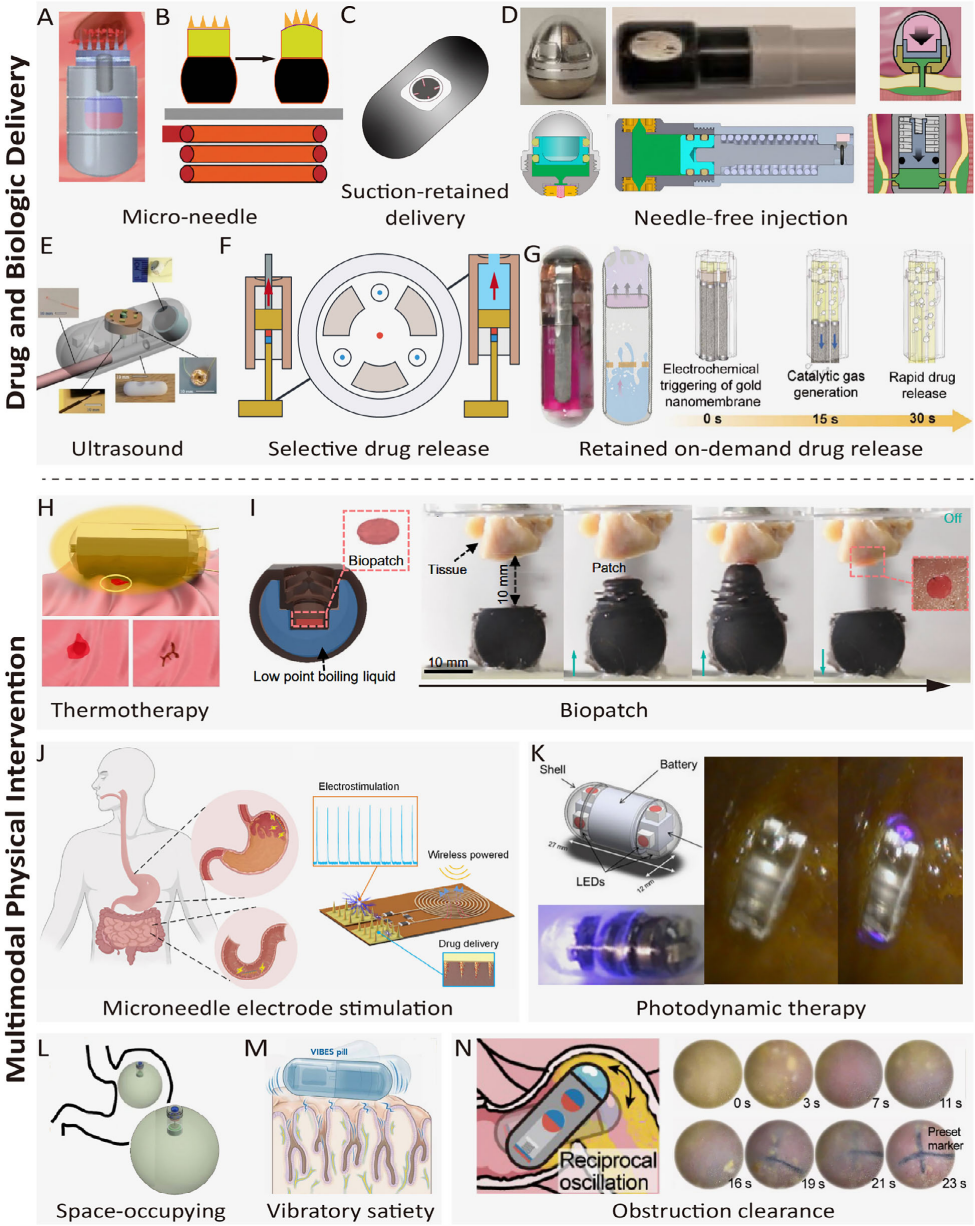
Therapeutic strategies of gastrointestinal capsule robots. (A) A magnetically driven dual‐layer microneedle patch for multisite targeted drug delivery at lesions. Reproduced with permission [34]. Copyright 2026, Springer Nature. (B) A magnetothermal phase‐change‐actuated reversible microneedle system, in which evaporation and condensation of a low‐boiling‐point liquid enable microneedle insertion and retraction. Reproduced with permission [118]. Copyright 2024, National Academy of Sciences. (C) A pH‐triggered negative‐pressure attachment and microneedle injection system for tissue anchoring, submucosal injection, and sustained release in specific intestinal environments. Created by the authors using Adobe Illustrator, based on the working principle of Ref [132]. (D) A needle‐free liquid microjet injection system that achieves localized and precise drug delivery through high‐pressure microjet ejection. Reproduced with permission [133]. Copyright 2024, Springer Nature. (E) A focused‐ultrasound‐enhanced drug delivery system that promotes deep tissue penetration through cavitation effects. Reproduced with permission [135]. Copyright 2021, MDPI. (F) A rotatable multi‐chamber therapeutic capsule in which a lead‐screw motor enables switching between biopsy and drug‐delivery modes, while a piston extrudes drugs or hydrogel precursors. Created by the authors using Adobe Illustrator, based on the working principle of Ref [136]. (G) A long‐term gastric‐retentive on‐demand drug‐release system that uses electrochemical triggering and a gas‐generating piston for rapid emergency drug release. Reproduced with permission [137]. Copyright 2025, American Association for the Advancement of Science. (H) A therapeutic capsule integrating a heating membrane and phase‐change materials for thermal coagulation hemostasis and staged drug release. Reproduced with permission [138]. Copyright 2025, MDPI. (I) An acoustothermal biopatch delivery system that uses phase‐transition‐driven expansion to press a tissue patch onto the lesion surface for in situ adhesive repair. Reproduced with permission [114]. Copyright 2024, Springer Nature. (J) A wirelessly powered electrostimulation‐assisted microneedle delivery system that integrates drug‐loaded microneedle electrodes with localized electrical stimulation, enabling simultaneous tissue penetration, drug release, and electrophysiological modulation for applications such as neuromodulation or tissue functional recovery. Reproduced with permission [139]. Copyright 2026, Wiley. (K) A photodynamic therapy capsule that integrates a specific‐wavelength light source to exploit endogenous porphyrins within Helicobacter pylori as natural photosensitizers, generating reactive oxygen species under illumination for selective bacterial eradication without requiring exogenous photosensitizing agents. Reproduced with permission [141]. Copyright 2024, Springer Nature. (L) A magneto‐chemically triggered intragastric balloon system for weight‐loss therapy, in which an external magnetic field initiates an acid‐base reaction within the capsule, generating gas for in situ balloon inflation and achieving space‐occupying intervention in the gastric cavity. Reproduced with permission [143]. Copyright 2016, Springer Nature. (M) A vibrating ingestible bioelectronic stimulator (VIBES) that mechanically stimulates gastric wall mechanoreceptors to mimic postprandial gastric distension, activating the vagal nerve‐brain axis to induce satiety‐associated neuroendocrine responses and significantly reducing food intake without surgical implantation or pharmacological intervention. Reproduced with permission [144]. Copyright 2023, American Association for the Advancement of Science. (N) A mechanical clearance capsule that utilizes the robot's mechanical output to transmit kinetic energy to the surrounding medium, inducing agitation and fluidization effects that alter the physical state of impacted chyme, thereby achieving non‐invasive fragmentation and clearance of gastrointestinal obstructions. Reproduced with permission [76]. Copyright 2026, American Association for the Advancement of Science.

Microneedle‐mediated injection physically breaches the mucus and epithelial barriers that constrain conventional oral bioavailability, and representative systems differ mainly in their triggering mechanisms and the reversibility of needle deployment. Chen et al. integrated a dual‐layer microneedle patch and a permanent magnet within a capsule robot, as shown in Figure 6A [34]. The internal circuitry releases a protective shield via an electrothermal triggering mechanism, and the microneedles loaded with epinephrine are subsequently driven into the lesion tissue by an external magnetic field, enabling multi‐site targeted drug delivery. This system is capable of completing both visual diagnosis and precision therapy within a single examination procedure. To explore alternative control schemes for microneedle actuation, Sun et al. developed a modular micro‐robot based on the combination of magnetic droplets and a rigid outer shell, integrating an injection module containing a low‐boiling‐point liquid and a microneedle patch, as shown in Figure 6B [118]. An external radiofrequency magnetic field induces a magnetothermal effect in the ferrofluid, causing the low‐boiling‐point liquid within the sealed chamber to vaporize and expand. The pneumatic pressure generated by this phase transition pushes the sealing membrane upward and drives the microneedles into the tissue. Upon deactivation of the radiofrequency field, the liquid condenses and the microneedles retract automatically, providing a reversible and minimally invasive intraluminal delivery pathway. Pursuing a battery‐free route, Sarker et al. constructed a system integrating a vacuum chamber, a pH‐sensitive membrane valve, a tissue attachment structure, and a drug injection needle, as shown in Figure 6C [132]. Upon triggering by small‐intestinal pH environment, the membrane dissolves and initiates negative pressure, drawing intestinal wall tissue into the attachment chamber while simultaneously driving microneedles into the submucosal layer to complete injection. After the attachment device separates from the main body, it can remain lodged in the intestinal wall for extended periods, enabling sustained drug release without any batteries or electronic components. Taken together, these designs trade external addressability against onboard autonomy and single‐shot operation against reversible actuation, while stable tissue anchoring and controllable injection under peristalsis remain the principal limitations of microneedle‐based delivery.

Beyond solid microneedles, needle‐free and physically enhanced schemes pursue mucosal penetration without a persistent rigid structure. Arrick et al. developed a cephalopod‐inspired needle‐free liquid microjet system with axial and radial injection modules suited to different GI morphologies, as shown in Figure 6D [133]. The drug is pressurized to tens of bars and ejected at high velocity through micrometer‐scale nozzles, directly penetrating the mucosa to accomplish needle‐free injection. This approach replaces solid microneedle structures with a high‐pressure fluid instantaneous penetration mechanism. Addressing deep‐tissue delivery, Stewart et al. integrated a miniaturized focused ultrasound transducer and a drug delivery channel within a capsule, as shown in Figure 6E [134, 135]. When the drug flows through the focal zone of the transducer, ultrasound excitation induces localized microbubble oscillation and rupture to achieve targeted release. The mechanical effects generated by cavitation transiently enhance cell membrane permeability and significantly improve drug penetration into deep tissue layers under non‐contact conditions. Although both strategies avoid the mechanical and retention risks of solid needles, they depend on high pressure or external energy input, so precise control of dose and penetration depth remains their main constraint.

While the preceding systems focus on overcoming the mucosal barrier, another line of work shifts attention toward broadening therapeutic functionality and extending the operational time window, either by integrating multiple functions within one device or by enabling long‐term retained, on‐demand release. Li et al. engineered a rotatable multi‐chamber cylindrical structure in which individual chambers can be configured as either biopsy needles or drug storage units, as shown in Figure 6F [136]. Driven by an onboard miniaturized lead‐screw stepper motor, the system enables functional switching between multiple biopsies and on‐demand drug delivery, and in the delivery mode the motor actuates a piston to extrude medication or hydrogel precursors over the wound surface for in situ treatment. This multi‐chamber rotary architecture enhances functional flexibility and repeated‐operation capability, and its biopsy function also connects to the diagnostic strategies discussed in Section 4.1. Targeting chronic disease management and acute episodes, Huang et al. constructed a long‐term gastric‐retentive capsule integrating a biogalvanic cell, an electrically triggered gold membrane, and an effervescent‐driven piston, as shown in Figure 6G [137]. Following oral administration, the capsule can remain retained in the stomach for up to 10 weeks with stable drug storage. When an external wireless command or an internal sensor detects abnormal physiological signals, the gold membrane dissolves and allows citric acid and sodium bicarbonate to react and generate high‐pressure CO_2_ gas, which drives the piston to rapidly release emergency medication. This combination of long‐term retention and on‐demand release offers new perspectives for remote early warning and automated drug administration in sudden‐onset conditions such as epilepsy and cardiac arrhythmias. These multifunctional and long‐acting systems gain integration and standby capability at the cost of greater structural and control complexity and a reliance on onboard power or external command.

#### 4.2.2 Physical, Bioelectronic, and Multimodal Interventions

Beyond drug delivery, capsule robots are increasingly being explored as platforms for physical, bioelectronic, and multimodal interventions, including thermal therapy, electrophysiological modulation, photodynamic therapy, metabolic intervention, and mechanical clearance.

For wound management, thermal capsules combine hemostasis with controlled release. Xu et al. integrated a miniaturized electromagnetic coil, a heating membrane, and phase‐change materials with different melting points within a capsule robot, as shown in Figure 6H [138]. Rapid and uniform heating enables thermal coagulation hemostasis at wound surfaces. By stepwise adjustment of the heating voltage, paraffin layers with progressively higher melting points are sequentially melted to trigger multi‐stage drug release, so that hemostasis and staged delivery are executed within a single device. Sharing the phase‐change actuation principle of Sun's design but aimed at tissue repair, Hao et al. integrated an elastomeric chamber doped with Fe3O4 nanoparticles together with a biological tissue patch within a soft robot, as shown in Figures 6I and 4D [114]. Focused ultrasound induces vaporization and expansion of the internal low‐boiling‐point liquid, which pushes a piston to press the patch against the tissue surface for in situ adhesive repair. These thermal and phase‐change schemes enable effective sealing and hemostasis, but require careful confinement of the heating zone to avoid thermal injury to adjacent healthy tissue.

Introducing electrophysiological modulation, Zhao et al. encapsulated drugs within a microneedle electrode structure and proposed a combined minimally invasive strategy that integrates microneedle‐mediated drug delivery with localized electrical stimulation, as shown in Figure 6J [139]. The design enables simultaneous tissue puncture and drug delivery while applying localized electrical stimulation signals, introducing an electrophysiological mechanism beyond chemical therapy for applications such as neuromodulation or tissue functional recovery. As a combined modality, the coordination between electrical and chemical dosing and the in vivo effect of luminal stimulation still require systematic validation.

To address refractory GI infections, Orsini et al. integrated a light source of specific wavelength within the capsule, allowing the device to reside in the target gastric region and deliver sustained illumination, as shown in Figure 6K [140, 141]. The system exploits the endogenous porphyrins inherently present within Helicobacter pylori as photosensitizers, generating reactive oxygen species under illumination to achieve selective eradication of the pathogen without administering exogenous photosensitizing agents. The advantage of avoiding exogenous photosensitizers comes with a limited treatment range, because the effect is confined to the illuminated region and depends on sustained capsule residence at the target site.

For metabolic disease intervention, two surgery‐free strategies have been demonstrated. Do et al. proposed a magnetically controlled chemical reaction in which an external magnetic field initiates an acid‐base reaction inside the capsule, as shown in Figure 6L [142, 143]. The generated gas drives in situ balloon inflation within the gastric cavity to achieve space‐occupying weight‐loss therapy, enabling self‐deployment and inflation without surgery. Through an alternative mechanism, Srinivasan et al. developed a Vibrating Ingestible BioElectronic Stimulator (VIBES) that uses mechanical vibration to stimulate gastric wall mechanoreceptors and mimic the gastric distension that normally follows food intake, as shown in Figure 6M [144]. By activating the vagal nerve‐brain axis, the stimulus triggers satiety‐associated neuroendocrine responses, producing perceived fullness and significantly reducing food intake and weight gain, with no surgical implantation or pharmacological agent and natural excretion after GI transit. The two routes differ in reversibility and energy demand, since the space‐occupying balloon relies on a single triggered chemical reaction while the vibratory approach depends on sustained mechanical actuation and onboard power.

For mechanical obstruction within the digestive tract, Xiang et al. used the mechanical output of the robot to transmit kinetic energy to the surrounding medium, as shown in Figure 6N [76]. Through mechanical agitation and fluidization effects that alter the physical state of locally impacted chyme, the system achieves fragmentation and clearance of GI obstructions as a non‐invasive alternative for digestive tract clearance. Its effectiveness depends on the robot's force output and on the consistency of the impacted material, which constrains the range of obstructions it can address.

#### 4.2.3 Emerging Biohybrid Therapeutic Strategies

Beyond these physically and chemically driven modalities, the integration of magnetic micro/nanoparticles or magnetotactic bacteria within capsule robot platforms represents an emerging biohybrid therapeutic strategy. Magnetic particles can serve both as actuating elements for magnetically triggered drug release and as localized therapeutic agents through magnetothermal effects, whereas magnetotactic bacteria offer intrinsic self‐propulsion and magnetic navigation capabilities. Encapsulating these magnetic or bioactive agents within capsule systems could enable controllable and targeted therapeutic delivery within the GI tract, potentially combining the responsive actuation of magnetic materials, the environmental adaptability of biological agents, and the structural integration advantages of engineered capsule platforms [145, 146, 147, 148].

Overall, therapeutic capsule robots now span precision drug delivery and multimodal physical interventions, markedly improving the precision and controllability of intraluminal local therapy while extending their functional boundaries beyond chemical therapy toward physical and combined interventions. However, existing capsule robots remain largely confined to relatively mild interventional modalities, such as drug delivery, tissue adhesion, hemostasis, and localized physical stimulation, and are not yet capable of performing surgical‐grade operations in vivo, including polypectomy, tissue incision, suturing, or complex hemostatic procedures. The core challenges lie in limited actuation capability, insufficient force output, constrained real‐time manipulation precision, and the need for further improvements in safety and controllability. Looking ahead, with continued advances in high‐power micro‐actuation systems, reconfigurable structural designs, and closed‐loop control strategies, capsule robots may evolve from precision drug delivery tools into active interventional platforms equipped with minimally invasive surgical capabilities, ultimately realizing true diagnostic‐therapeutic integration and serving as viable alternatives to intraluminal minimally invasive surgery.

## 5 Technology Transfer and Commercialization

The technological translation and commercialization progress of capsule robots constitutes a critical indicator for assessing the maturity of this field in transitioning from laboratory prototypes to clinical applications. Over the course of more than two decades of sustained development, multiple categories of capsule robot products have been successfully commercialized and introduced into clinical practice, with their application scope progressively expanding from initial luminal visualization and inspection toward microenvironment sensing and monitoring, targeted therapeutic intervention, and digital medication adherence management (Table. 3).

**TABLE 3:** Technology transfer and commercialization.

Function	Categorization	Product / Series	Institution	Core Technology / Features	Technical Advantages	Limitations
Luminal Visualization & Structural Diagnosis	1.1 Passive Optical Endoscopy	PillCam Series [149]	Medtronic	Earliest commercialized globally	Mature commercial platforms; no sedation; broad visualization	Passive peristalsis‐dependent transit; no active control; no biopsy or therapy; reading burden
		CapsoCam Plus [150]	CapsoVision	360° panoramic imaging capsule		
		EndoCapsule EC‐10 [151]	Olympus	3D Track for anatomical localization		
		MiroCam [152]	IntroMedic	Human Body Communication for data transmission		
		OMOM HD [153]	Jinshan Science & Technology	Bi‐directional RF communication Real‐time adjustment of working parameters		
	1.2 Active Optical Endoscopy (Magnetic)	NaviCam Series [154]	Ankon Technologies	Permanent magnet + external magnetic field	Operator‐controlled targeted visualization; improved coverage; reduced blind spots	Requires external magnetic platform and dedicated setting; operator training and equipment cost
		OMOM Magnetic System [155]	Jinshan Science & Technology	Magnetically controlled version		
		MiroCam‐Navi [156]	IntroMedic	Handheld magnet‐controlled version		
		ds‐MCE [157]	Ankon Technologies	Manual threading combined with magnetic control		
		JIFU Standing System [158]	Jifu Medical / Zifu Medical	Standing position examination		
	1.3 Active Endoscopy (Self‐Propelled)	PillBot [159]	Endiatx	Remote‐controlled Self‐propelled	Active maneuverability within the lumen without large external magnetic actuation	Increased internal complexity and power consumption; limited clinical validation
	1.4 Non‐Optical 3D Mapping	C‐Scan [160, 161]	Check‐Cap	Ultra‐low dose X‐ray imaging 2D/3D mapping of the colon	Colonic screening with reduced or no bowel preparation; 2D/3D anatomical mapping	Ionizing radiation; limited soft‐tissue contrast; no biopsy or therapy
II. Microenvironment Sensing & Monitoring	2.1 Physicochemical Parameter Monitoring	MotiliCap	AnX Robotica	pH, pressure, temperature, gastric emptying, and small bowel transit time	Quantitative pH, pressure, temperature, and motility assessment; simple workflow	Limited to selected physiological parameters; no direct visualization or intervention
		SmartPill [162]	Medtronic			
		Bravo pH Capsule [163]	Medtronic	pH Affixed to the esophageal wall		
		e‐Celsius [164]	BodyCap	GI temperature		
		IntelliCap [165]	Medimetrics	pH and temperature		
	2.2 Gas Metabolite Monitoring	Atmo Gas Capsule [166, 167]	Atmo Biosciences	H_2_, CO_2_, O_2_, and temperature Microbial fermentation	Direct in situ gas profiling; assesses microbial fermentation and metabolic activity	Niche indications; clinical interpretation of multivariable gas signals not yet standardized
	2.3 Hemorrhage Detection	HemoPill [168, 169]	Ovesco Endoscopy AG	Hematin and blood	Rapid detection of acute GI bleeding markers	Detection only; limited localization; no therapeutic capability
	2.4 Pathological Sampling (Tethered)	Cytosponge [170]	Cyted	Esophageal cells	Minimally invasive cellular or secretion sampling; relatively low device complexity	Tether discomfort; limited spatial reach; generally single‐region sampling
		EnteroTracker [171]	EnteroTrack	Mucosal secretions		
		EsophaCap / EsoCheck [172, 173]	CapNostics / Lucid Diagnostics	Barrett's Esophagus and esophageal sampling		
	2.5 Pathological Sampling (Untethered)	RSS Capsule [115]	Biora Therapeutics	Aspirates liquid at target locations and physically locks	Site‐specific luminal liquid sampling with internal sealing	Limited sample volume; localization accuracy and sample integrity remain challenges
III. Targeted Intervention & Mechanical Therapeutics	3.1 Localized Luminal Drug Delivery	NaviCap [174]	Biora Therapeutics	Targeted release of liquid therapeutics in the colon	Targeted release at specific GI segments; improves local drug exposure	Limited payload; requires reliable localization or triggering
		IntelliCap [175]	Medimetrics	Targeted drug release		
	3.2 Mucosal Penetration / Macromolecule Injection	RaniPill [176, 177]	Rani Therapeutics	Deploy a degradable microneedle for painless injection	Enables oral delivery of biologics or macromolecules; avoids conventional injections	Single‐use mechanism; injection reliability, anchoring, and tissue safety require validation
		BioJet	Biora Therapeutics	Delivers biotherapeutics to the small intestine wall via liquid jet for systemic absorption		
	3.3 Physical Mechanical Stimulation	Vibrant [178]	Vibrant Gastro	Low‐frequency vibrations to stimulate intestinal mechanoreceptors, promoting peristalsis for constipation	Drug‐free therapy for motility‐related disorders; generally well tolerated	Indication‐specific; requires onboard power; long‐term efficacy and safety need validation
		Vibrabot [179]	Ankon Technologies	Physical vibrational stimulation treating functional slow transit constipation		
IV. Medication Adherence Monitoring	4.1 Adherence Monitoring	ID‐Cap System [180]	etectRx	Activated by gastric fluid to send a signal to an external reader confirming ingestion	Objective documentation of medication ingestion; supports digital health monitoring	Adds device complexity to oral drugs; privacy, acceptability, and reimbursement concerns
		Abilify MyCite [181]	Otsuka Pharmaceutical / Proteus Digital Health	Digital pill Activated by stomach acid and transmit signals to a patch and app		

In the domain of luminal visualization and structural diagnosis, passive optical capsule endoscopes represent the earliest commercialized and currently most widely adopted product category in clinical practice. The PillCam capsule endoscopy system from Medtronic, as one of the first globally commercialized capsule endoscopy systems, established the technological foundation and clinical application paradigm for this field [149]. Subsequently, multiple manufacturers have pursued continuous improvements in imaging capability and system functionality. For example, the CapsoCam Plus capsule endoscope from CapsoVision achieved 360‐degree panoramic imaging [150]. The EndoCapsule EC‐10 capsule endoscope from Olympus introduced 3D Track functionality for anatomical localization [151]. The MiroCam capsule endoscopy system from IntroMedic adopted Human Body Communication technology for data transmission [152]. The OMOM HD capsule endoscopy system from Jinshan Science and Technology enabled bi‐directional radiofrequency communication with real‐time adjustment of working parameters [153]. To overcome the navigational limitations imposed by passive peristalsis‐dependent transit, magnetically controlled active capsule endoscopes have also progressively entered the commercialization stage. For example, the NaviCam magnetic capsule endoscopy system from Ankon Technologies achieves controlled capsule locomotion through an external magnetic field [154]. The OMOM magnetic capsule endoscopy system from Jinshan Science and Technology and the MiroCam‐Navi magnetic capsule endoscopy system from IntroMedic provide magnetically controlled and handheld magnet‐controlled solutions, respectively [155, 156]. The ds‐MCE detachable string magnetic capsule endoscopy system from Ankon Technologies combines manual threading with magnetic control navigation, enabling complete examination of the esophagus, stomach, and small intestine [157]. Furthermore, the standing position examination system from JIFU Medical further expands the application scenarios of gastric magnetic‐controlled examination [158]. In the area of self‐propelled capsules, the PillBot endoscopic robot developed by Endiatx, a remote‐controlled self‐propelled capsule robot, is currently undergoing clinical trials [159]. In the non‐optical imaging domain, the C‐Scan capsule colonoscopy system developed by Check‐Cap employs ultra‐low dose X‐ray imaging technology to generate two‐dimensional and three‐dimensional anatomical images of the colon [160, 161]. This approach provides a potential alternative for colonic screening that eliminates the need for bowel preparation.

In the domain of microenvironment sensing and physiological monitoring, commercialized capsule products have expanded beyond optical visualization to include measurement of physicochemical parameters, metabolic gas detection, hemorrhage identification, and pathological sampling. For physicochemical parameter monitoring, several ingestible capsules integrate multi‐sensor systems to characterize gastrointestinal motility and environmental conditions. For instance, the SmartPill capsule from Medtronic incorporates pH, pressure, and temperature sensors within a single platform, enabling quantitative assessment of gastric emptying time and small bowel transit time for the clinical evaluation of GI motility disorders [162]. The MotiliCap capsule from AnX Robotica provides comparable multi‐parameter monitoring capabilities targeting similar physiological indices. In contrast, the Bravo pH capsule from Medtronic adopts a different deployment strategy by attaching directly to the esophageal wall, enabling prolonged ambulatory esophageal pH monitoring without nasogastric catheterization [163]. For core body temperature monitoring, the e‐Celsius capsule from BodyCap continuously records gastrointestinal temperature during transit [164]. The IntelliCap capsule from Medimetrics further integrates pH and temperature sensing with onboard drug delivery functionality, representing an example of combined sensing and therapeutic capability within a single capsule platform [165]. In the area of metabolic gas monitoring, the Atmo Gas Capsule from Atmo Biosciences measures concentrations of hydrogen, carbon dioxide, and oxygen along with temperature within the gastrointestinal lumen [166, 167]. This technology enables direct in situ characterization of microbial fermentation activity, which has traditionally been assessed only through indirect breath testing methods. For intraluminal hemorrhage detection, the HemoPill capsule from Ovesco Endoscopy AG detects hematin and blood within the GI tract, providing a potential tool for rapid identification of acute gastrointestinal bleeding events [168, 169]. Beyond real‐time sensing, pathological sampling capsules have also entered commercialization, including both tethered and untethered designs. Among tethered systems, the Cytosponge from Cyted collects esophageal cells upon retrieval of an expandable sponge for subsequent cytological analysis [170]. The EnteroTracker from EnteroTrack enables collection of mucosal secretions from targeted intestinal segments, while the EsophaCap/EsoCheck from CapNostics is designed for esophageal cell sampling with applications in Barrett's esophagus screening [171, 172, 173]. In the untethered category, the RSS capsule from Biora Therapeutics aspirates intestinal liquid at target locations and seals the collected sample within internal chambers for subsequent ex vivo analysis [115]. Collectively, these sensing and sampling capsules demonstrate the expanding role of ingestible devices in physiological monitoring and minimally invasive diagnostic assessment within the gastrointestinal tract.

In the domain of targeted intervention and therapeutics, commercialized capsule products have covered multiple pathways including localized drug delivery, mucosal penetration injection, and mechanical stimulation therapy. For example, the NaviCap targeted drug delivery capsule from Biora Therapeutics and the IntelliCap drug delivery capsule from Medimetrics both support targeted drug release in specific regions of the digestive tract [174, 175]. For macromolecular drug delivery, the RaniPill ingestible drug delivery capsule from Rani Therapeutics achieves injection by deploying a degradable microneedle within the small intestine, while the BioJet liquid jet injection system from Biora Therapeutics delivers biotherapeutics to the small intestinal wall via liquid jet for systemic absorption [176, 177]. In the area of physical stimulation therapy, the Vibrant capsule for chronic constipation from Vibrant Gastro employs low‐frequency vibrations to stimulate intestinal mechanoreceptors and promote intestinal peristalsis for the treatment of chronic constipation [178]. The Vibrabot capsule from Ankon Technologies is also based on vibrational stimulation principles for the treatment of functional slow transit constipation [179].

Additionally, medication adherence monitoring, as a recently emerged digital healthcare application, has also achieved initial commercialization. For example, the ID‐Cap ingestible event marker system from etectRx is activated upon contact with gastric fluid and transmits a signal to an external receiver to confirm whether the medication has been ingested [180]. Abilify MyCite, co‐developed by Otsuka Pharmaceutical and Proteus Digital Health, utilizes a miniaturized sensor activated by stomach acid, a body‐surface patch, and a mobile application to achieve objective documentation and remote monitoring of patient medication‐taking behavior [181].

Despite the growing number of commercialized capsule products, current clinical adoption remains concentrated in relatively low‐risk functions such as luminal visualization, motility monitoring, pH sensing, temperature monitoring, medication adherence tracking, and simple targeted drug delivery, whereas several important clinical needs remain insufficiently addressed, including controllable whole‐tract navigation, accurate real‐time localization, reliable tissue biopsy, active hemostasis, polypectomy, perforation closure, and closed‐loop diagnosis‐guided therapy. This gap indicates that the commercialization of capsule robots has not yet fully matched the broader clinical demand for integrated diagnostic‐therapeutic and interventional intraluminal platforms. In addition to technical maturity, cost represents a critical factor governing clinical adoption and market penetration. Compared with conventional passive capsule endoscopes, active capsule robots often require onboard power units, localization modules, wireless communication circuits, micro‐actuators, drug delivery mechanisms, and external magnetic actuation or wireless control platforms, all of which increase manufacturing and procedural costs. Magnetically controlled systems in particular may rely on bulky external actuation equipment and dedicated examination settings, limiting accessibility in primary care and resource‐limited environments. Customized fabrication, complex assembly, sterilization, regulatory validation, and reimbursement uncertainty further affect large‐scale deployment. Future commercialization will therefore need to prioritize not only diagnostic and therapeutic performance, but also low‐cost materials, scalable manufacturing, reusable external actuation platforms, simplified clinical workflows, and clearer reimbursement pathways. Balancing performance, safety, and affordability will be essential for transforming capsule robots from advanced prototypes into widely deployable clinical products [24].

Overall, the commercialization of capsule robots has established a preliminary yet diversified product landscape spanning diagnosis, monitoring, therapy, and digital health management. Passive optical capsule endoscopes and GI motility monitoring capsules have entered the mature stage of clinical application, while magnetically controlled active capsule endoscopes, targeted drug delivery systems, and physical stimulation therapeutic capsules are progressively completing the transition from proof‐of‐concept to clinical deployment. However, compared with the rich repertoire of functional prototypes demonstrated at the laboratory stage, the functional integration level of currently commercialized products remains relatively limited, with most products focusing on a single diagnostic or therapeutic function and not yet achieving system‐level integration of combined diagnostics and therapeutics. Furthermore, active control schemes that are highly dependent on external magnetic field equipment still face challenges in terms of system portability and operational simplicity, while the commercialization of self‐propelled capsules and advanced therapeutic capsules remains at an early stage. Looking ahead, with continued advances in miniaturized actuation technologies, high‐density functional integration, and intelligent closed‐loop control capabilities, commercialized capsule robots are expected to progressively transcend the boundaries of single‐function products and evolve toward comprehensive intraluminal intelligent medical platforms that integrate detection, diagnosis, and therapy.

## 6 Current Limitations

Despite the significant progress achieved by capsule robots in recent years across driving mechanisms, diagnostic functionalities, therapeutic strategies, and commercial applications, a considerable technological gap remains before the realization of an intraluminal intelligent medical platform equipped with autonomous navigation, multimodal perception, and closed‐loop control capabilities. Current systems continue to face multiple critical bottlenecks in material structure, energy supply, environmental perception, and system‐level integration. Accordingly, this section summarizes the core challenges and future development directions from four dimensions: materials and structural design, energy supply and actuation systems, perception and intelligence, and system‐level functional integration.

### 6.1 Materials and Structural Design

Current capsule robot shell designs remain predominantly based on rigid polymers or metals, exhibiting notable deficiencies in both traversability and tissue compatibility when navigating the highly tortuous and dynamically deforming luminal environments of the digestive tract. Concurrently, the development of intelligent anchoring mechanisms represents an urgent challenge. Prolonged physiological monitoring or sustained therapeutic intervention requires capsules to resist the strong peristaltic emptying forces of the intestine and remain stably stationed at target regions. However, how to ensure anchoring reliability while simultaneously avoiding tissue ischemia or mucosal damage remains the core engineering difficulty in this direction.

### 6.2 Energy Supply and Actuation Systems

Energy supply constitutes the fundamental bottleneck constraining the functional integration density and operational duration of capsule robots. Conventional button cell batteries occupy nearly half of the capsule's internal volume, yet typically support only several hours to roughly half a day of operation in commercial capsule endoscopes, which is barely sufficient to cover small bowel transit and falls short of the timescales required for whole‐tract examination or prolonged in situ monitoring. This constraint reflects a fundamental tension among capsule volume, energy capacity, and functional complexity: as additional sensing, communication, and active actuation modules are integrated, the available volume for the battery is progressively squeezed, while the overall power demand increases. Continuous functions such as imaging and wireless data transmission dominate baseline consumption, whereas active actuation events such as micro‐pump compression or puncture release can transiently draw substantially higher peak power. Achieving efficient energy reception, rectification, and management within miniaturized volumes, particularly when the system must switch dynamically between peak driving demands and ultra‐low‐power standby modes, continues to pose formidable hardware design challenges. At the actuation level, although existing magnetic driving systems possess advantages in terms of controllable degrees of freedom, several practical issues remain prominent. External magnetic platforms typically rely on bulky permanent‐magnet manipulators or large electromagnetic coil arrays that occupy dedicated examination rooms and entail considerable equipment cost, which limits their accessibility in primary care settings. In addition, the magnetic field strength attenuates rapidly with distance from the external source, reducing the effective driving force on capsules located deep within the abdomen, and the spatial resolution of magnetic control remains coarse compared with that of conventional endoscopic instruments.

### 6.3 Perception and Intelligence

At the perception level, the imaging and sensing systems of current capsule robots are largely confined to single modalities, making it difficult to characterize the multi‐dimensional pathological information of digestive tract tissue comprehensively. At the intelligence level, existing AI‐assisted capsule endoscopes predominantly rely on offline post‐processing by external receivers or cloud‐based analysis, rendering real‐time intracorporeal lesion recognition and response unattainable.

### 6.4 System‐level Functional Integration

From the perspective of system‐level integration, the majority of current capsule robots focus exclusively on a single diagnostic or therapeutic function, with a considerable gap remaining before the realization of genuine diagnostic‐therapeutic integration. However, the magnetic coupling interference encountered when using a single external magnetic field to independently control multiple magnetic micro‐robots in a fluidic environment, along with fundamental challenges in inter‐capsule communication and anti‐collision path planning, place this direction firmly at an early proof‐of‐concept stage.

**FIGURE 7:**
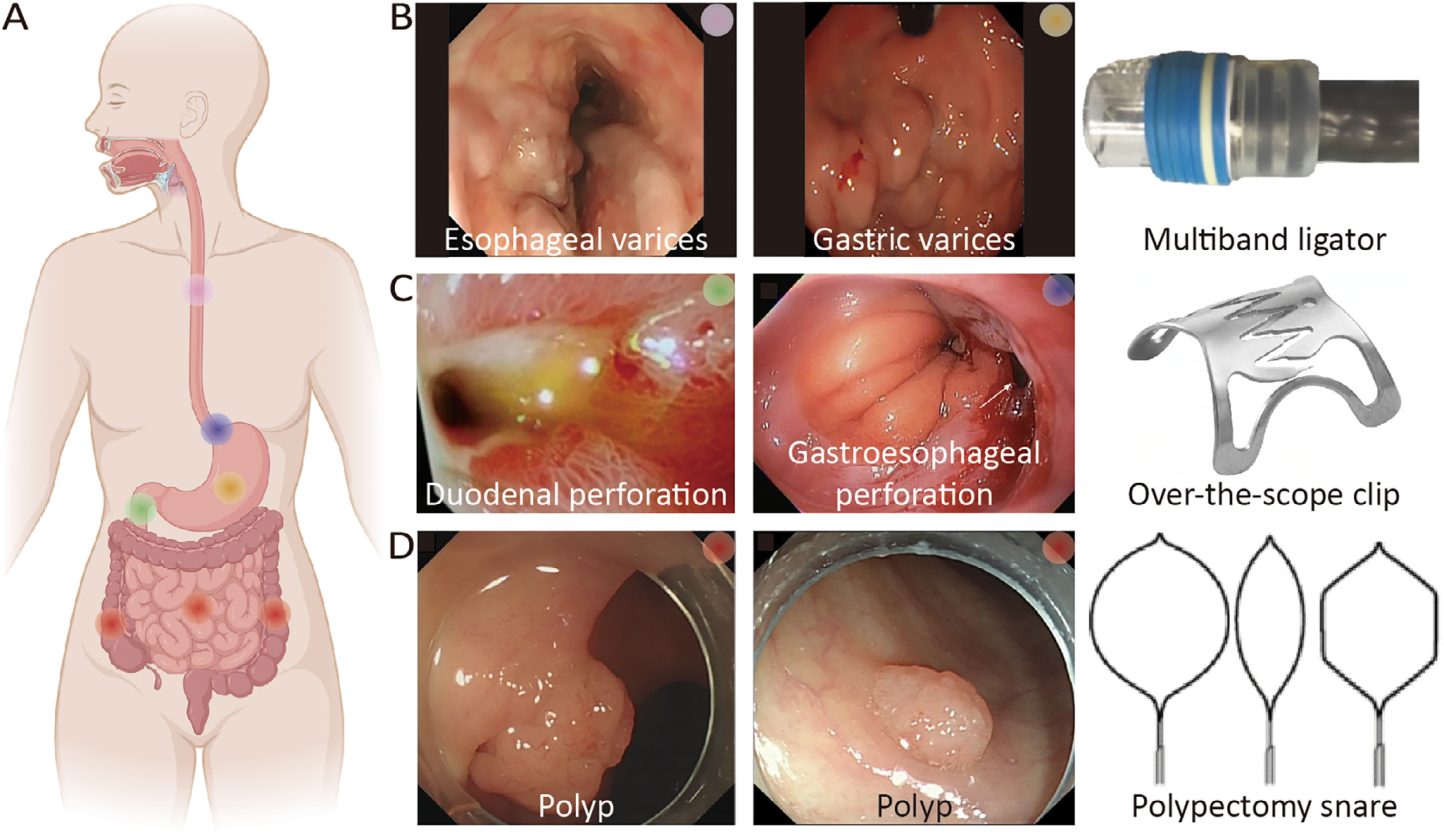
Representative gastrointestinal pathologies that currently require conventional endoscopic intervention and remain beyond the capabilities of capsule robots. (A) Anatomical overview of the gastrointestinal tract with colored markers indicating the locations of representative pathologies. Created by the authors using Adobe Illustrator. (B) Endoscopic images of esophageal varices and gastric varices, alongside the corresponding therapeutic device, a multiband ligator used for endoscopic variceal band ligation. Reproduced with permission [184, 185]. Copyright 2019, Springer Nature; Copyright 2025, Baishideng Publishing Group. (C) Endoscopic images of duodenal perforation (left) and gastroesophageal perforation (right), alongside an over‐the‐scope clip used for endoscopic closure of full‐thickness gastrointestinal defects. Reproduced with permission [186, 187]. Copyright 2023, EpiSmart; Copyright 2020, Baishideng Publishing Group. (D) Endoscopic images of gastrointestinal polyps, alongside a polypectomy snare used for endoscopic polyp resection. Reproduced with permission [189, 190]. Copyright 2023, Oxford Academic; Copyright 2016, Korean Society of Gastrointestinal Endoscopy.These conditions collectively illustrate the current functional gap between conventional endoscopic platforms and capsule robot systems, particularly in mechanical hemostasis, tissue closure, and lesion resection, thereby highlighting key directions for future capsule robot development.

It is further worth noting that current GI clinical practice still encompasses multiple key pathological scenarios that remain beyond the capabilities of capsule robots. As illustrated in Figure 7A, pathologies including esophageal and gastric varices, GI perforations, and polyps are widely distributed across various segments of the digestive tract and still rely primarily on conventional endoscopic intervention. Among these, the band ligation treatment of esophageal and gastric varices requires stable tissue anchoring and precise manipulation of multiband ligators (Figure 7B) [182, 183, 184, 185]. The closure and repair of GI perforations rely on the mechanical closure capability of over‐the‐scope clips for full‐thickness defects (Figure 7C) [186, 187, 188]. The complete resection of polyps requires the coordinated execution of precise snare positioning, tightening, and electrocautery (Figure 7D) [189, 190]. The transient force output, multi‐degree‐of‐freedom tool coordination, and stable mechanical anchoring demanded by these surgical‐grade operations constitute the most prominent functional gap between current capsule robots and conventional endoscopic platforms. From a long‐term perspective, bridging this gap represents the core of the paradigm shift for capsule robots from passive observation and lightweight intervention toward active surgical‐grade intervention. With continued advances in materials science, micro‐nano fabrication, wireless energy transfer, and artificial intelligence technologies, future capsule robots are expected to progressively overcome existing technological bottlenecks and play an increasingly important clinical role in the screening, monitoring, and minimally invasive treatment of selected digestive tract diseases.

## 7 Future Directions and Emerging Trends

Building on the limitations identified above, this section outlines the principal directions along which capsule robots are expected to evolve. The discussion follows the same four dimensions of materials, energy, perception, and system‐level integration, and further highlights two emerging frontiers that have attracted considerable recent attention: artificial intelligence‐driven autonomous operation and magnetically actuated capsule swarms. Across these directions, clinical translation will depend on aligning engineering advances with unmet clinical needs and value‐centered design [24].

### 7.1 Soft, Programmable, and Multi‐Material Architectures

The development of shell materials is expected to move away from rigid encapsulation toward flexible and soft‐body architectures. Elastomeric materials embedded with magnetic microparticles can replace conventional rigid shells, allowing capsules to undergo compliant deformation under magnetic field guidance and thereby adapt to narrow folds and complex curved luminal segments. In parallel, a range of intelligent anchoring strategies is being explored to keep capsules stationed at target regions against strong peristaltic forces, including expandable support structures, shape memory alloy‐based self‐adaptive mechanisms, and bioinspired micro‐adhesion surfaces. Beyond single‐material solutions, multi‐material synergistic fabrication strategies that incorporate rigid‐flexible composites and functionally graded architectures hold promise for achieving a more favorable balance between structural support and tissue protection. Looking further ahead, emerging concepts such as programmable magnetization and microscale soft robots may substantially expand the functional repertoire of ingestible robotic systems [191, 192, 193].

### 7.2 Advanced Power Solutions and High‐Power‐Density Actuation

Overcoming the energy bottleneck will require a transition from conventional button cell batteries toward battery‐free power solutions. Promising approaches include radiofrequency wireless power transfer, extracorporeal focused ultrasound energy transmission, and biofuel cells that exploit the electrochemical reactions of gastric acid. These strategies have the potential to fundamentally relax endurance limitations and liberate internal volume for additional functional components. The remaining challenge lies in achieving efficient energy reception, rectification, and management within a miniaturized volume, particularly when the system must switch dynamically between peak driving demands and ultra‐low‐power standby modes. At the actuation level, future breakthroughs are anticipated in high‐power‐density micro‐actuators, including more efficient miniaturized electromagnetic coils, piezoelectric drivers [194, 195], and magnetic‐elastic coupling mechanisms. The objective is to deliver greater force output and more refined motion control within progressively smaller volumes.

### 7.3 On‐Board Artificial Intelligence and Autonomous Operation

Two complementary trends are reshaping the role of intelligence in capsule robots. The first is autonomous navigation. Conventional capsule operation depends heavily on manual manipulation by trained physicians, which limits accessibility and reproducibility. Machine vision and reinforcement learning have been investigated to enable partial to full autonomy in magnetic capsule control, with demonstrations progressing from simulation environments to ex vivo validation in animal tissue, while in vivo autonomous control of the capsule itself remains an open challenge [196, 197]. The second trend is the migration of artificial intelligence from external post‐processing into the capsule itself. Existing AI‐assisted capsule endoscopes rely largely on offline analysis by external receivers or cloud platforms, which precludes real‐time intracorporeal response. The integration of lightweight neural network chips or low‐power neuromorphic processors directly onto the capsule circuit board would enable an intracorporeal closed loop of real‐time image recognition, autonomous decision‐making, and triggered execution. A first proof of concept of this paradigm has already been demonstrated, in which a capsule carrying an on‐board deep neural network detected colorectal polyps in real time during an in vivo porcine study [13]. Upon automatic identification of a bleeding site or suspicious lesion, such a capsule could in principle trigger drug release or hemostatic intervention without external operation, marking the transition from a passive diagnostic tool toward an autonomous interventional platform. In parallel, the miniaturized integration of deep‐tissue imaging modalities such as optical coherence tomography, photoacoustic imaging, and micro‐ultrasound transducer arrays would provide depth‐resolved three‐dimensional information on the GI wall without the discomfort of conventional endoscopic ultrasound. The continued miniaturization of capacitive micromachined ultrasonic transducer arrays is moving capsule‐integrated high‐resolution ultrasound from concept verification toward engineering realization.

### 7.4 Multi‐Capsule Cooperation, Capsule Swarms, and Surgical‐Grade Intervention

At the system level, the long‐term objective is genuine diagnostic‐therapeutic integration on a single platform, combining high‐resolution imaging, multi‐parameter sensing, targeted drug delivery, and minimally invasive intervention within a single ingestion. Realizing this goal requires not only synergistic advances across materials, energy, and perception, but also high‐density module integration within an extremely limited volume.

One near‐term strategy is multi‐capsule cooperation, in which several capsule modules are magnetically connected or coordinated to expand functional space and distribute tasks [198, 199, 200, 201, 202]. Existing studies in this direction are better described as modular multi‐capsule collaboration rather than true swarm systems, since only a small number of modules are physically connected or magnetically coupled. Whether multi‐capsule cooperation can ultimately deliver distributed imaging, sampling, or therapeutic capabilities remains to be shown, alongside challenges in coupling reliability, traversal of curved intestinal segments, and coordination under peristaltic disturbance. Another more forward‐looking direction is capsule swarm‐enabled intervention. True swarm behavior requires large numbers of relatively independent agents that collectively form reconfigurable patterns through shared physical fields. At the capsule scale, this remains largely conceptual, whereas swarm‐like collective behaviors have been clearly demonstrated at the micro‐ and nanorobot scale, where magnetic agents can be reconfigured into chain, vortex, ribbon, or liquid‐state formations under external magnetic fields [146]. These works provide the methodological foundation for future capsule systems, in which a capsule may serve not only as a therapeutic device itself but also as a carrier or release depot for large numbers of microscale agents. Such swarm‐ or micromotor‐based luminal strategies have shown initial in vivo feasibility: fluoroscopy‐guided magnetic microswarms have eradicated biofilms in biliary stents [145], photoacoustic‐guided ingestible microrobots have achieved intestinal navigation and prolonged retention in vivo [203], and degradable capsules releasing biohybrid algae motors have improved intestinal distribution of therapeutic payloads after oral administration [204]. Translating these principles to true capsule‐scale swarms could ultimately enable cooperative imaging, regional sampling, and distributed multi‐site intervention, although independent control under a shared field, inter‐capsule communication, collision avoidance, and safe retrieval or excretion remain major open challenges.

The most demanding frontier concerns surgical‐grade intervention. As illustrated in Figure 7, key procedures including variceal band ligation, perforation closure, and polypectomy still depend on conventional endoscopy because they require transient high force output, multi‐degree‐of‐freedom tool coordination, and stable mechanical anchoring. Bridging this gap represents the core of the paradigm shift for capsule robots from passive observation and lightweight intervention toward active surgical‐grade intervention. With continued advances in materials, micro‐ and nanofabrication, wireless energy transfer, artificial intelligence, and coordinated robotic control, future capsule robots may progressively overcome these bottlenecks and assume an increasingly important clinical role in the screening, monitoring, and minimally invasive treatment of selected gastrointestinal diseases.

## 8 Conclusion

This review has provided an overview of the key technological framework of capsule robots for the gastrointestinal tract from a systems engineering perspective. Over the course of more than two decades of sustained development, significant progress has been achieved across multiple technical dimensions, advancing from concept verification toward clinical application. Shell material systems have evolved from early single rigid encapsulation toward multi‐material architectures reconciling mechanical support, tissue protection, and environmental adaptability. Actuation technologies have formed a diversified landscape encompassing passive peristalsis, magnetic actuation, multi‐legged propulsion, and wireless power‐driven mechanisms. Diagnostic capabilities have expanded from conventional white‐light imaging to multimodal in situ detection, including multi‐parameter biochemical sensing, spectrally enhanced imaging, and three‐dimensional structural reconstruction. Therapeutic functions have extended from simple drug delivery toward multiple intervention modalities including microneedle injection, photodynamic therapy, thermal hemostasis, and mechanical obstruction clearance. In parallel, multiple categories of commercialized products have been deployed across GI examination, motility monitoring, and targeted therapy scenarios. Nevertheless, critical technological bottlenecks persist in rigid‐flexible hybrid fabrication of materials and structures, battery‐free high‐power‐density solutions for energy and actuation, multimodal fusion and edge AI closed‐loop integration for perception and intelligence, and system‐level diagnostic‐therapeutic integration. The long‐term developmental objective of capsule robots is to progressively evolve from early swallowable imaging devices toward intraluminal intelligent medical platforms integrating autonomous navigation, intelligent diagnosis, precision therapy, and safe excretion. The realization of this objective necessitates deep interdisciplinary convergence across robotics, materials science, microelectronic engineering, artificial intelligence, and clinical medicine. With continued breakthroughs in key enabling technologies and the ongoing advancement of system‐level integration capabilities, capsule robots are expected to play an increasingly important clinical role in the future screening, long‐term monitoring, and minimally invasive treatment of gastrointestinal diseases.

## Author Contributions

**Zengsheng Li**: investigation, writing – original draft. **Zhongguo Ren**: investigation, writing – original draft. **Xianfeng Xia**: writing – review and editing. **Mengmeng Sun**: conceptualization, writing – review and editing, supervision, resources, funding acquisition. **Yiliang Lin**: writing – review and editing. **Tianlong Li**: writing – review and editing, supervision, funding acquisition. **Philip Wai Yan Chiu**: writing – review and editing, supervision. **Wei Peng Yong**: writing – review and editing. **Kai Fung Chan**: supervision, writing – review and editing, funding acquisition.

## Conflicts of Interest

The authors declare no conflicts of interest.

## Data Availability

The data that support the findings of this study are available from the corresponding author upon reasonable request.
